# Dietary Behaviours and Association with Nutritional Status Among Malaysian School-Based Adolescents: Findings from Adolescent Health Survey 2022

**DOI:** 10.3390/nu18111833

**Published:** 2026-06-05

**Authors:** Lay Kim Tan, Guey Yong Chong, Shi Hui Cheng, Sumarni Mohd Ghazali, Chee Cheong Kee

**Affiliations:** 1 Sector for Biostatistics & Data Repository, Office of NIH Manager, National Institutes of Health, Ministry of Health Malaysia, Shah Alam 40170, Selangor, Malaysia; kee@moh.gov.my; 2 Department of Nutrition, Faculty of Medicine and Health Sciences, Universiti Putra Malaysia, Serdang 43400, Selangor, Malaysia; 3 Research Centre of Excellence, Nutrition and Non-Communicable Diseases, Universiti Putra Malaysia, Serdang 43400, Selangor, Malaysia; 4 School of Biological and Environmental Sciences, Faculty of Science and Engineering, University of Nottingham Malaysia, Semenyih 43500, Selangor, Malaysia; shihui.cheng@nottingham.edu.my; 5 Biomedical Epidemiology Unit, Special Resource Centre, Institute for Medical Research, Ministry of Health Malaysia, Shah Alam 40170, Selangor, Malaysia; sumarni.mg@moh.gov.my

**Keywords:** dietary behaviours, nutritional status, adolescents, AHS 2022, Malaysia

## Abstract

**Background/objective:** This study determined the prevalence of dietary behaviours and examined their relationship with nutritional status among Malaysian school-based adolescents. **Methods:** Data from 33,523 adolescents who participated in the Adolescent Health Survey (AHS 2022) were analysed. Multiple logistic regression was employed to assess associations between dietary behaviours and nutritional status, adjusting for potential confounders. **Results:** Our findings demonstrated a double burden of malnutrition, with 6.8% stunting, 8.3% thinness, and 30.5% overweight/obese. High prevalence of inadequate daily intake of fruit and vegetables (FV) (83.9%) and insufficient daily dairy consumption (62.7%) was observed. Approximately one-third of adolescents reported frequent fast-food consumption (i.e., at least one day per week) (35.4%) and daily intake of carbonated soft drinks (32.4%). Daily carbonated soft drink consumption was associated with higher odds of overweight/obesity (aOR:1.11; 95% CI:1.04–1.20), highlighting the need to prioritise the public health strategies aimed at reducing sugar intake. Conversely, inadequate FV intake (aOR:0.88; 95% CI:0.81–0.95) and frequent fast-food consumption (aOR:0.87; 95% CI:0.82–0.94) were associated with lower odds of overweight/obesity, while insufficient daily dairy intake was associated with lower odds of thinness (aOR:0.83; 95% CI:0.73–0.94). These negative associations should be interpreted cautiously due to possible reverse causality and reporting bias. **Conclusions:** The findings highlight the importance of strengthening nutrition education and the food environment among Malaysian adolescents.

## 1. Introduction

Adolescence, spanning ages 10 to 19, is defined as the second decade of life by the World Health Organization (WHO) and is characterised by profound physical, psychological and social changes [1]. Globally, adolescents make up approximately one-quarter of the total population, around 1.8 billion, with 90% of them residing in low- and middle-income countries (LMICs) [2,3].

Establishing healthy dietary habits during adolescence is crucial for optimal growth and development of organs, cognitive functions, and physiological systems, which lay the foundation for healthy adulthood and contribute to the achievement of sustainable societies, aligning with the United Nations Sustainable Development Goal 3 (Good Health and Well-Being). Despite its importance, adolescent nutrition is increasingly challenged by the rising double burden of malnutrition (DBM), particularly in LMICs. Recent global data highlights the presence of both under- and overnutrition among adolescents. A pooled analysis involving 3663 population-representative studies involving 222 million participants spanning from 1990 to 2022 reported substantial global burdens of both undernutrition and obesity among adolescents [4]. In 2022, 77.0 million girls and 108 million boys were thin or underweight, while 65.1 million girls and 94.2 million boys were obese [4], with thinness declining in Southeast Asia, sub-Saharan Africa, and South Asia, but increasing in Yemen, Niger, and Myanmar. Adolescent stunting data are limited globally. Stunting prevalence in Ethiopia and India ranged from 21.8% to 29.6% [5,6,7], while long-term decline in stunting was observed among Chinese adolescents due to targeted rural nutrition programs [8]. Despite reductions in thinness and stunting, obesity sharply increased, especially in Polynesia, Micronesia, the Caribbean, Brunei, and Chile [4]. More recently, the WHO reported that over 390 million children and adolescents worldwide aged 5–19 years were overweight in 2022, including 160 million living with obesity [9].

Unhealthy eating behaviours drive both undernutrition and overnutrition. A meta-analysis of Global School-Based Student Health Surveys (GSHSs) across Africa, Asia, Oceania, and Latin America reported that 34.5% of the adolescents consumed fruit less than once per day, 20.6% consumed vegetables less than once per day, 42.8% drank carbonated soft drinks at least once per day, and 46.1% consumed fast food at least once per week [10]. While global data on total dairy intake among adolescents are lacking, evidence from multiethnic United States populations showed a decline in dairy consumption among adolescents compared to childhood, with this often falling below the WHO’s recommendation of three cups per day, with non-Hispanic Black adolescents having the lowest and non-Hispanic White adolescents [11] the highest total dairy intake.

Adherence to healthy dietary behaviours, including adequate intake of fruits and vegetables (FV), and dairy products, plays an important role in preventing micronutrient deficiencies and diet-related non-communicable diseases among adolescents. For example, adequate FV intake has been significantly associated with a lower prevalence of thinness among Pakistani adolescents and a decrease in body mass index (BMI) among the school-aged adolescents in Tanzania [12,13]. However, no significant association was reported between FV intake and reduced risk of stunting among Iranian children and early adolescents [14]. Evidence from multiple countries shows that higher dairy consumption is linked to improve linear growth and reduced risk of underweight and stunting in children and adolescents [15,16,17].

On the other hand, overconsumption of carbonated beverages and fast foods, both of which are considered to be unhealthy dietary behaviours, has sharply increased among adolescents and raised global concern about associated nutrition-related health risks. However, findings on this association have been mixed. For instance, frequent fast-food intake was associated with higher risk of stunting among school-going adolescent girls in Northwest Ethiopia [18], but in a study among Indian adolescents, the negative of this association was found [19]. An Indonesian longitudinal study reported that frequent consumption of fast food and frequent consumption of soft drinks were each independently associated with higher odds of following a BMI trajectory that progressed from overweight to obesity among children and adolescents aged 6–18 years [20]. These inconsistent findings highlight a clear evidence gap, emphasising the need for further studies in diverse populations. Consistent with these global trends, Malaysia is also experiencing a growing double burden of malnutrition and unhealthy dietary behaviours among adolescents. The most recent Malaysian Adolescent Health Survey (2022) reported that among Malaysian school-going adolescents, 16% met the recommended FV intake, 33% consumed carbonated soft drinks daily, 10.6% consumed fast food at least three times per week, and 23.2% reported milk and dairy consumption [21]. In this same population, 6.8% were stunted, 8.3% were thin, while 16.2% were overweight and 14.3% were obese [21]. However, despite the growing concern over adolescent nutrition, data on the relationship between dietary behaviours and nutritional status in this population remain limited.

While dietary behaviours frequently cluster with other lifestyle habits to influence broader metabolic pathways, isolating the specific contributions of distinct dietary habits remains critical for targeted public health interventions. Therefore, the present study aimed to describe dietary behaviours including adequacy status, specifically thinness, overweight/obesity, and stunting among Malaysian adolescents using data from the AHS 2022. Additionally, we examined associations between dietary behaviours and nutritional status to inform targeted nutrition intervention in this population.

## 2. Materials and Methodology

### 2.1. Study Design and Study Population

The present study utilised data from the Adolescent Health Survey (AHS) 2022. The survey was a nationwide cross-sectional study conducted by the Ministry of Health Malaysia in collaboration with the Ministry of Education Malaysia. The original survey aimed to provide nationally representative data on health behaviours and related factors among Malaysian adolescents aged 13 to 18 years attending secondary school. The AHS 2022 was approved by the MOH Medical Research and Ethics Committee (MREC) (MREC Ref: 21-157-58261) with additional permission obtained from the Ministry of Education Malaysia at all levels. Both students and their parents or legal guardians provided written informed consent prior to participation. Participant confidentiality and anonymity were protected at all stages of data handling and reporting. For the present study, new ethical approval was obtained from MREC to perform a secondary analysis using data from the original survey (i.e., AHS 2022) stored at the NIH-Data Repository System (MREC Ref: 25-04263-OOF). This study was reported in accordance with the Strengthening the Reporting of Observational Studies in Epidemiology (STROBE) guideline for cross-sectional studies (Supplementary File S1).

### 2.2. Sampling Design

A two-stage stratified cluster sampling design was applied to obtain a nationally representative sample. The sample size was determined using the single-proportion formula based on prevalence estimates from the previous AHS study [22], with 95% confidence level, margin of error between 1% and 5%, and design effect of 2 to account for the cluster sampling. The estimated sample size was subsequently increased by 20% to allow for potential non-response.

In the first stage, 240 secondary schools were selected from 2798 eligible schools using probability proportional to size sampling across 13 states and three Federal Territories. A total of 16 secondary schools were randomly selected from each of the 13 states, whereas a smaller cluster of 8 schools was selected from each of the three Federal Territories (Kuala Lumpur, Labuan, and Putrajaya) due to their smaller geographical size and school density. Sampling weights were subsequently applied to ensure national representativeness. In the second stage, classes within the selected schools were systematically chosen, and all students in the selected classes were invited to participate. The final sample comprised approximately 36,000 adolescents, representing an average of 2250 students per state or Federal Territory. Further details of the sampling procedure have been described in the technical report [21].

### 2.3. Data Collection and Quality Assurance

Data collection was conducted between June and July 2022 by 34 trained field teams, each supervised by a field supervisor. A one-week centralised training session was organised to standardise procedures and ensure data quality. Students completed optical mark recognition (OMR) questionnaires during school hours under supervision. Quality control was maintained through daily field verification, supervisory oversight, and central coordination by the Institute for Public Health (IPH). All OMR forms were verified and processed at the IPH Data Processing and Quality Centre, where data were checked for completeness and consistency prior to analysis [21,23].

### 2.4. Study Variables

Study variables were grouped into dependent and independent measures, with additional covariates included to control for potential confounding. The dependent variables were indicators of nutritional status, whereas the independent variables comprised dietary behaviours. Covariates comprised sociodemographic characteristics and lifestyle-related factors that have been shown in previous studies to influence adolescent nutritional status outcomes and were therefore adjusted for in multivariable analyses.

#### 2.4.1. Dependent Variables

The dependent variables were indicators of nutritional status, namely BMI for age Z-score (BAZ) and height-for-age Z-score (HAZ), computed according to the WHO 2007 Growth Reference for adolescents aged 5 to 19 years [24]. Nutritional status was categorised into thin, normal, overweight, or obese based on BAZ, and stunted or normal/tall based on HAZ, according to WHO-recommended cut-offs (thinness < −2 SD; overweight > +1 SD; obesity > +2 SD for BAZ; stunting < −2 SD for HAZ).

#### 2.4.2. Independent Variables

The independent variables comprised measures of dietary behaviours that reflected both healthy and unhealthy eating behaviours. Healthy dietary indicators included FV intake and daily dairy product intake. Adequate fruit intake is defined as consuming at least two servings of fruit per day, while adequate vegetable intake is defined as consuming at least three servings of vegetables per day. Individuals who meet both criteria—≥2 servings of fruit and ≥3 servings of vegetables daily—are considered to have adequate FV intake. Meanwhile, daily dairy product intake is defined as adequate when consumed at least twice per day. Unhealthy dietary indicators consisted of fast-food intake, considered frequent when consumed on one or more days per week, and carbonated soft drink intake, considered frequent when consumed at least once per day. In addition to these single indicators, combined dietary behaviours were created to examine the potential synergistic effect between healthy and unhealthy behaviours on nutritional status, including the joint categories of FV intake with fast-food intake, FV intake with carbonated soft drink intake, daily dairy product intake with fast-food intake, and dairy product intake with carbonated soft drink intake.

#### 2.4.3. Covariates

Sociodemographic variables included sex (male and female), age group (13 to 16, 17 to 18 years), ethnicity (Malay, Chinese, Indian, Bumiputra Sabah and Sarawak and others) and parental marital status (married and living together, married but living apart and divorce/widow/separated), while lifestyle factors covered smoking status (yes or no), alcohol consumption (yes or no), physical activity (active or inactive) and sedentary behaviour (yes or no). Physical activity was assessed using the GSHS module and categorised according to WHO recommendations, where adolescents engaging in at least 60 min of moderate-to-vigorous activity on five or more days per week were considered active. Sedentary behaviour referred to time spent sitting or in screen-based leisure activities for three or more hours per day outside of schoolwork. Comorbidity was represented by self-reported hunger status, which served as a proxy indicator of food insecurity and was categorised as ‘never’, ‘sometimes or rarely’, and ‘most of the time’ or ‘always’.

Although the WHO defines adolescents as individuals aged 10 to 19 years [1], this study included participants aged between 13 and 18 years to reflect the structure of the Malaysian secondary school system. In Malaysia, secondary education generally comprises students aged 13 to 17 years; however, a small number of students aged 12 years may enrol in transition classes prior to entering Form 1, while others aged 18 years may remain in Form 5 due to late school enrolment, grade repetition, or extended examination schedules. This approach ensures that all adolescents enrolled in secondary schools are represented in the analysis, consistent with the national sampling frame of the AHS 2022. The inclusion criteria align with the Malaysia Education Blueprint 2013–2025 and the official Ministry of Education Malaysia description of the secondary education pathway [25].

### 2.5. Statistical Analysis

All statistical analyses were conducted using IBM SPSS Statistics version 31.0.0.0 (117) (SPSS IBM, New York, NY, USA) with the Complex Samples procedures to account for the two-stage stratified cluster design and sampling weights, ensuring nationally representative estimates. Descriptive statistics were used to summarise sociodemographic characteristics, lifestyle factors, dietary behaviours, and nutritional status of the study population. Categorical variables were expressed as weighted percentages with 95% confidence intervals (CI).

Associations between dietary behaviours and nutritional status were examined using complex logistic regression analysis. Two models were constructed: Model 1 adjusted for sociodemographic variables (age, sex, ethnicity, and parental marital status) and lifestyle factors (smoking and alcohol consumption); Model 2 further included hunger status, physical activity and sedentary behaviour.

Variable coding and reference categories followed National Health and Morbidity Survey (NHMS) standards. Nutritional status outcomes were analysed as dichotomous variables: overweight/obese (BAZ ≥ +1 SD) versus normal (−2 SD ≤ BAZ < +1 SD; reference), thinness (BAZ < −2 SD) versus normal, and stunting (HAZ < −2 SD) versus normal/tall stature (HAZ ≥ −2 SD; reference). Dietary pattern variables were coded as binary indicators: fruit intake (reference = adequate ≥ 2 servings/day; inadequate < 2 servings/day), vegetable intake (reference = adequate ≥ 3 servings/day; inadequate < 3 servings/day), daily dairy product intake (reference = adequate ≥ 2 time (s)/day; inadequate < 2 time(s)/day), fast-food intake (≥1 day (s)/week; reference = ≤1 day/week), and carbonated soft drink intake (≥1 time(s)/day; reference = ≤1 time/day). Combined dietary behaviour variables were constructed to assess potential interactions between healthy and unhealthy behaviours, specifically between FV intake with fast-food intake, FV intake with carbonated soft drink intake, daily dairy product intake with fast-food intake, and dairy product intake with carbonated soft drink intake. Exploratory two-way interaction analyses were performed for these combined dietary behaviour variables.

Covariates were entered as categorical variables with the following reference categories: female (sex), 17 to 18 years (age group), others (ethnicity), married and living together (parental marital status), no (smoking and alcohol consumption), never (hunger status), inactive (physical activity), and no (sedentary behaviour). Adjusted odds ratio (aOR) and 95% CI were reported for each model. A *p*-value of less than 0.05 was considered statistically significant. Data completeness was ensured through centralised data verification procedures prior to analysis. Participants with missing data on key variables, including dietary behaviours, anthropometric outcomes, or covariates used in regression models, were excluded from the analysis (complete-case approach). The proportion of missing data was low and unlikely to introduce significant bias; therefore, no imputation techniques were performed.

Model predictive ability was evaluated using the receiver operating characteristic (ROC) curve and the classification table, with details presented in Supplementary File S2, Table S1. ROC curve statistics demonstrated satisfactory discrimination for all models, while variance inflation factor analysis indicated no evidence of multicollinearity, supporting model stability and appropriate model specification.

## 3. Results

### 3.1. Sociodemographic Factors, Lifestyle Characteristics and Nutritional Status

Table 1 presents the sociodemographic and lifestyle characteristics of 33,523 Malaysian school-based adolescents aged between 13 and 18 years. Half of the adolescents are male (50.0%), indicating an equal distribution by gender. Most of the adolescents were aged between 13 and 16 years (81.4%), Malay (63.0%), and had parents who were married and living together (79.5%). The majority were non-smokers (91.0%) and non-drinkers (92.6%). Our findings show that for every 100 Malaysian school-based adolescents, 68 had never gone hungry, 67 reported three hours or more of sedentary behaviour daily, whereas only 21 met the criteria for being physically active.

**Figure 1:**
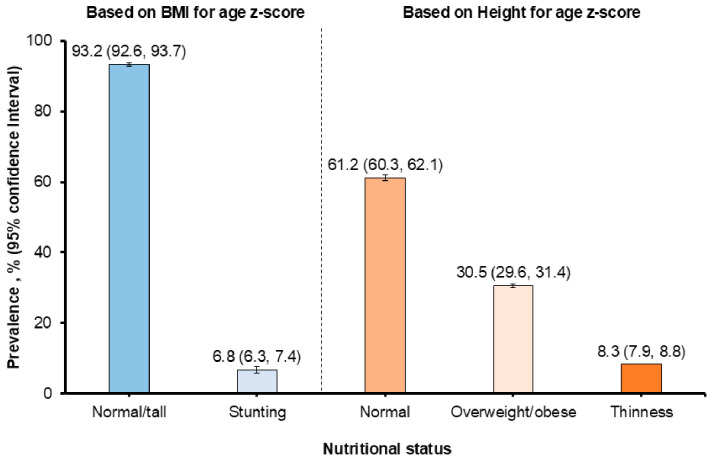
Prevalence of nutritional status among Malaysian school-based adolescents.

In terms of nutritional status (Figure 1), we observed that 6.8% of adolescents were stunted based on HAZ. Regarding BMI-for-age, the majority (61.2%) had a normal BAZ, followed by 30.5% who were overweight/obese, and 8.3% who were classified as thin. These findings reflect the coexistence of DBM among Malaysian school-based adolescents.

### 3.2. Dietary Behaviours Among Malaysian Adolescents

**Figure 2:**
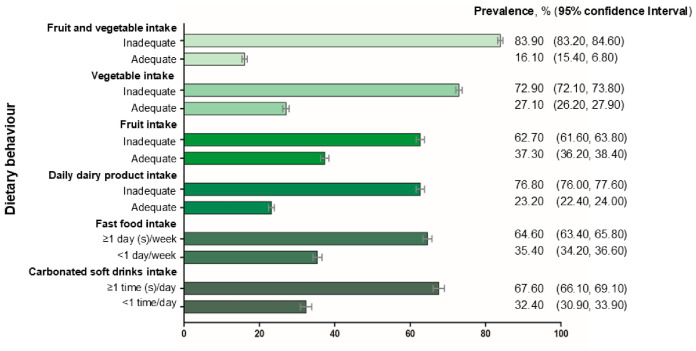
Prevalence of dietary behaviours among Malaysian school-based adolescents.

Figure 2 illustrates the prevalence of dietary behaviours among Malaysian school-based adolescents. Overall, a high proportion (83.9%) of adolescents did not achieve the recommended daily intake of FVs. When examined separately, 62.7% and 72.9% of adolescents failed to meet the recommended daily intake of the respective fruit and vegetable groups. Similarly, insufficient intake of daily dairy products was observed in 76.8% of the adolescents. We further observed a high prevalence of unhealthy dietary behaviours among the Malaysian school-based adolescents. Approximately 64.6% of adolescents consumed fast food at least once per week, while 67.6% reported daily intake of carbonated soft drinks, with the healthiest category as the reference group.

Supplementary File S2, Table S2 presents the prevalence of combined dietary behaviours among Malaysian school-based adolescents. A substantial proportion of the adolescents reported inadequate FV intake combined with frequent fast-food consumption (30.2%), while 25.4% reported inadequate FV intake in conjunction with daily soft drink consumption. In contrast, only a small proportion of the adolescents achieved adequate FV intake with no fast-food consumption (10.9%) or no daily soft drink intake (9.1%). With respect to daily dairy product intake, nearly one-third of the adolescents (28.9%) reported inadequate dairy consumption combined with frequent fast-food intake, while 21.6% reported inadequate daily dairy product intake in conjunction with daily soft drink consumption.

### 3.3. Associations Between Dietary Behaviours and Nutritional Status

**Figure 3:**
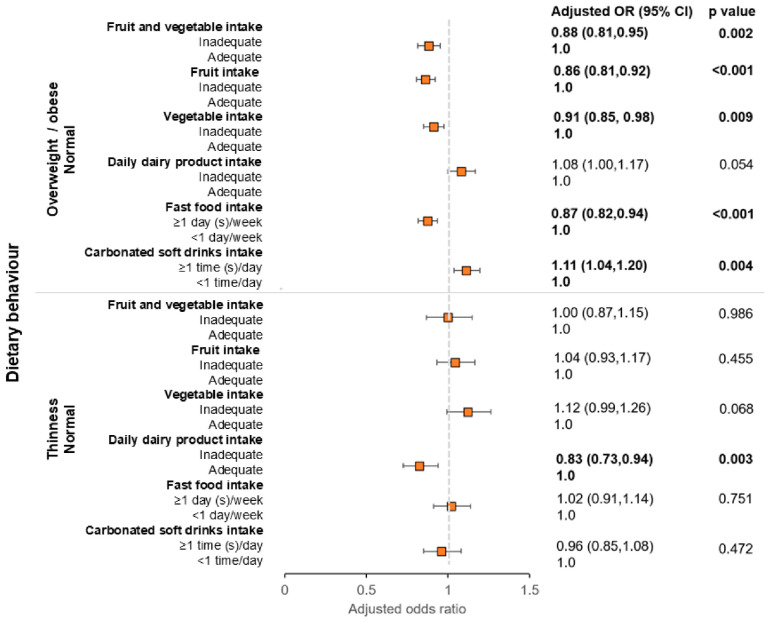
Associations between dietary behaviours and BMI for age z-score (BAZ), adjusted for age, sex, race, parent’s marital status, smoking status, alcohol status, hunger status, physical activity, and sedentary behaviours. Abbreviations: ORs: odds ratio; CI: confident interval.

Figure 3 and Supplementary File S2, Table S3 present the associations between dietary behaviours and BAZ among Malaysian school-based adolescents. School-based adolescents who did not meet the recommended daily intake of FVs were associated with lower odds of overweight/obese (aOR = 0.88; 95% CI: 0.81–0.95). Similar negative associations were observed in separate analyses for inadequate fruit intake (aOR = 0.86; 95% CI: 0.81–0.92) and inadequate vegetable intake (aOR = 0.91; 95% CI: 0.85–0.98). Unhealthy dietary behaviours were also associated with nutritional status. Frequent consumption of fast food was associated with lower odds of overweight/obese (aOR = 0.87; 95% CI: 0.82–0.94), whereas daily intake of carbonated soft drinks increased the risk of overweight/obese (aOR = 1.11; 95% CI: 1.04–1.20). Inadequate daily dairy intake was associated with lower odds of thinness (aOR = 0.83; 95% CI: 0.73–0.94) (Figure 3 and Supplementary File S2, Table S3).

We further performed the combined analysis to examine whether the effect of inadequate FV intake/daily dairy products on BAZ changes when it co-occurs with another unhealthy dietary behaviour—frequent fast-food intake or daily carbonated soft drink intake (Supplementary File S2, Table S4). When investigating inadequate FV intake combined with non-frequent fast-food intake, our data demonstrated significant association with lower odds of overweight/obesity (aOR: 0.85; 95% CI: 0.77–0.94) (Supplementary File S2, Table S4). In the combined analysis of inadequate FV intake and carbonated soft drink intake, both groups—with or without daily carbonated soft drink intake—showed an association with lower odds of overweight/obesity (aOR_IFVI + CSD ≥ 1_: 0.76; 95% CI: 0.67–0.85; aOR_IFVI + CSD < 1_: 0.80; 95% CI: 0.72–0.89) (Supplementary File S2, Table S4). The combination of adequate FV intake with daily carbonated soft drink intake showed a significant association with lower odds of overweight/obesity (aOR: 0.80; 95% CI: 0.69–0.93) (Supplementary File S2, Table S4), indicating that regardless of the adequacy of fruit and vegetable consumption, concurrent carbonated soft drink intake did not shift the direction of the association, unexpectedly suggesting a lower likelihood of overweight/obesity across all combined categories. This counterintuitive finding highlights the potential influence of residual confounding factors, such as overall energy intake or dietary under-reporting among overweight/obese individuals. Nevertheless, findings from the combined analysis should be interpreted cautiously, as they may reflect reporting bias, reverse causality, or other unmeasured behavioural factors.

When inadequate daily dairy intake was combined with frequent fast-food intake, a significant positive association with overweight/obesity was observed (aOR: 1.21; 95% CI: 1.09–1.34) (Supplementary File S2, Table S4). In contrast, this combined pattern was associated with lower odds of thinness (aOR: 0.83; 95% CI: 0.70–0.98) among school-based adolescents (Supplementary File S2, Table S4). These observations indicated that this specific dietary pattern is strongly associated with an upward shift in BMI.

**Figure 4:**
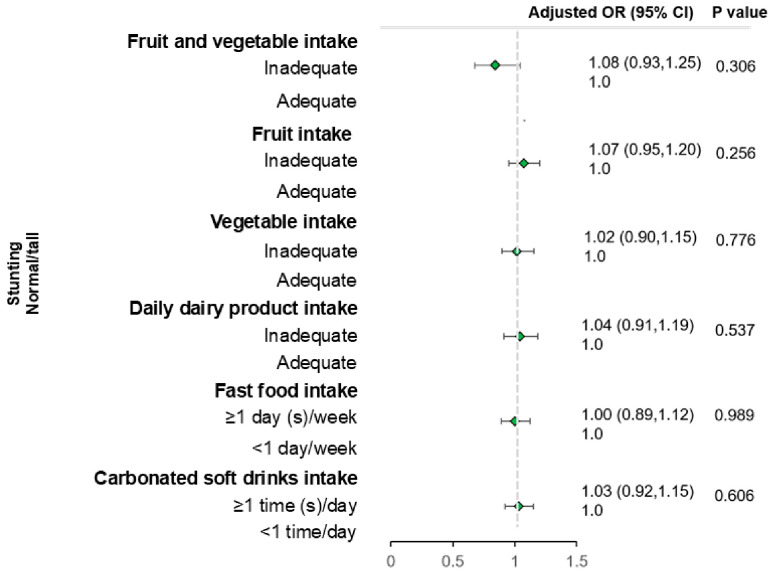
Associations between dietary behaviours and stunting, adjusted for age, sex, race, parents’ marital status, smoking status, alcohol status, hunger status, physical activity and sedentary behaviour. Abbreviations: OR: odds ratio; CI: confident interval.

Figure 4 and Supplementary File S2, Table S3 summarise the lack of significant associations between dietary behaviours and stunting among Malaysian school-based adolescents. First, no significant association between inadequate FV intake and stunting (aOR: 1.08; 95% CI: 0.93–1.25) was observed. A subsequent separate analysis demonstrated no associations between fruit intake (aOR: 1.07; 95% CI: 0.95–1.20) and vegetable intake (aOR: 1.02; 95% CI: 0.90–1.15) with stunting among school-based adolescents. Inadequate daily dairy product intake was not associated with stunting (aOR: 1.04; 95% CI: 0.91–1.19). We further observed no associations between unhealthy dietary behaviours and stunting, i.e., frequent fast-food intake (aOR: 1.00, 95% CI: 0.89–1.12) and carbonated soft drink intake (aOR: 1.03, 95% CI:0.92–1.15). Further combination analysis showed no significant association with stunting (Supplementary File S2, Table S4). Overall, we did not observe a significant association between dietary indicators with stunting.

## 4. Discussion

The present study highlights the coexistence of undernutrition and overnutrition, reflecting the DBM among Malaysian school-based adolescents. We further observed a high prevalence of unhealthy dietary behaviours in this population, including inadequate intake of FV, insufficient daily consumption of dairy products, frequent fast-food consumption, and daily intake of carbonated soft drinks. Our analyses further revealed that inadequate FV intake and frequent fast-food consumption were negatively associated with overweight/obesity, whereas daily consumption of carbonated soft drinks increased the risk of overweight/obesity. Additionally, insufficient daily dairy intake was negatively associated with thinness. No significant associations were found between these dietary behaviours and the risk of stunting.

Our findings on the double DBM among Malaysian adolescents align with growing evidence from both national and international studies showing that undernutrition and overnutrition often coexist in the same population in LMICs [26,27,28]. In our sample, we observed three times higher prevalence of overweight/obesity compared with thinness and stunting—a pattern that echoes the finding from the recent nationwide survey, i.e., NHMS 2024, where the prevalence of overweight/obesity is 28%, whilst 8.1% and 11.1% of the Malaysia adolescents were stunted and underweight, respectively [29]. Malaysia’s overweight/obesity burden appears comparable or higher than many neighbouring countries in South Asia [28]; the persistent undernutrition suggests that the nutrition transition is at an advanced stage, but socioeconomic disparities (e.g., rural vs. urban, household food security) still maintain undernutrition pockets. However, detailed socioeconomic indicators such as household income and parental education were not available in the AHS 2022 dataset; therefore, the direct contribution of socioeconomic status could not be comprehensively evaluated in the present study. Given this context, our results strengthen the case for integrated public health policies that simultaneously address under- and overnutrition: including healthier school food environments, regulation of sugar-sweetened beverages and ultra-processed foods, nutrition education and food literacy, and targeted support for undernourished subgroups.

These patterns may partly reflect Malaysia’s status as an upper-middle-income country with a relatively higher Human Development Index and national income—conditions commonly associated with increasing obesity prevalence [30]. Rapid socioeconomic development has accelerated the nutrition transition, marked by a shift from traditional diets and physically active lifestyles toward more ‘Westernised’ dietary behaviours (calorie-dense processed foods, higher sugar and fat intake), along with more sedentary behaviours.

In fact, national data have documented substantial changes in Malaysia’s food supply over recent decades including increases in wheat (+56.5%), sugar and sweeteners (+23.9%), meat (+49.3%), fish and seafood (+38.7%), and eggs (+55.7%), alongside a decrease in rice (−23.7%) and the plant-to-animal protein ratio over time [31]. These transitions in food environment and consumption may likely contribute to the observed DBM among adolescents. However, additional factors such as (i) lifestyle affected by psychological, biological, relationship, community and society influences, (ii) urbanisation and (iii) socioeconomic disparities are also likely to play important roles. However, they are not directly examined in the present study.

The low prevalence of healthy dietary behaviours (adequate FV intake, daily dairy product intake) and high prevalence of unhealthy dietary behaviours (frequent consumption of carbonated soft drinks and fast-food) observed in the present study were observed in many studies from different parts of the world. For instance, a meta-analysis of GSHS conducted between 2008 and 2015 found that about 34.5% of adolescents consumed fruit less than once per day and 20.6% consumed vegetables less than once per day; moreover, 42.8% drank carbonated soft drinks at least once per day and 46.1% reported fast-food consumption at least once per week [10]. Meanwhile, a large multinational study covering 49 LMICs documented that the prevalence of adolescents who met the WHO recommendation of adequate FV intake was below 30% [32]. Collectively, these data suggest widespread disparity in the prevalences of dietary inadequacy or unhealthy dietary behaviours among youths. This disparity may reflect country-specific differences: dietary guidelines, local food environments, accessibility and affordability of healthy foods, cultural food habits, and possibly socioeconomic factors. In addition to dietary behaviours alone, emerging evidence highlights the importance of considering the combined effects of lifestyle factors, particularly sleep and nutrition, on somatic health outcomes. A recent study demonstrated that dietary patterns interact with sleep habits in shaping body composition and cardiovascular indicators with healthy eating and structured meal patterns associated with more favourable body composition, while unhealthy behaviours such as energy drink consumption and late-night eating were linked to poorer outcomes, including lower fat-free mass and higher BMI [33]. While the present study recognises these complex interrelationships, data regarding concurrent lifestyle factors, e.g., sleep patterns, were unavailable for analysis within the secondary dataset used. Consequently, this highlights a critical gap in current national-level assessments. Future research is warranted to comprehensively investigate the synergistic effects of dietary habits and broader lifestyle factors to better understand their collective impact on adolescent metabolic health and body composition.

Furthermore, regarding traditional indicators such as BMI, recent evidence highlights the importance of identifying phenotypes such as normal weight obesity (NWO), characterised by normal BMI but excess body fat. A recent study among young adults demonstrated that NWO was associated with higher visceral fat and unfavourable lifestyle behaviours [34], further emphasising the importance of considering body composition and lifestyle-related factors when interpreting nutritional status outcomes. While the clinical relevance of these behavioural clusters and phenotypic variations is clear, they could not be investigated in the present study due to the inherent constraints of the secondary dataset, which lacks concurrent sleep data and advanced body composition metrics. Consequently, this represents a distinct gap in our current findings. Future nationwide research utilising multi-module assessments and body composition analysis is warranted to bridge these gaps and provide a more nuanced understanding of adolescent health in Malaysia. In Malaysia, the unhealthy dietary behaviours among adolescents mirrored those observed in adults [35,36], which may reflect dietary habits established during childhood. Parents’ influence on child dietary behaviours [37] further emphasises the critical role of the parents or caregivers in educating and instilling healthy dietary behaviours during early life and adolescence. Parents or caregivers with higher nutrition literacy are more likely to positively influence their children’s dietary behaviours [38], as they tend to make healthier food choices and are less susceptible to the pervasive influence of food advertising across television and social media platforms. Additionally, excessive screen time has been associated with unhealthy dietary behaviours in children and adolescents [39], highlighting the importance of parental monitoring and structured screen time limits to encourage healthier dietary habits among adolescents. Taken together, context-specific interventions that equip parents with nutrition literacy can serve as measurable strategies to ensure that healthy dietary behaviours are instilled in children and adolescents and sustained into adulthood, ultimately contributing to public health improvement and the development of a healthier nation.

Low adherence to healthy dietary behaviours—not complying with dietary guideline recommendations in relation to food group consumption to achieve all nutrients and energy required—could influence the clinical outcomes in a person in the long term [36,40]. In the present study, we observed that daily intake of a carbonated soft drink increased the risk of overweight and obesity among the Malaysian school-based students. Over the past decade, the increasing trend of daily carbonated soft drinks (at least one a day) from 29.3% to 32.4% among the Malaysian school-based students is alarming [41,42]. Furthermore, carbonated soft drink consumption was associated with poor mental health among Malaysian adolescents, with 1.3 higher odds of having depression and 1.4 higher odds of attempting suicide [43]. We found that consumption of carbonated soft drinks had an association with nutrition status, such as overweight and obesity. A pooled analysis involving 107 countries that participated in the Global School-Based Student Health Survey (2009–2017), the European Health Behavior in School-Aged Children study (2017–2018), and the US Youth Risk Behavior Survey (2019) reported a significant association between daily soft drink consumption and overweight and obesity, with an increased odds of 1.14 among school-going adolescents [44]. Our neighbouring country, Indonesia, which shares cultural similarities, also reported that carbonated soft drink consumption was associated with 1.58 times higher odds of being overweight among Indonesian adolescents [20]. The findings underscore the necessity of a proactive, school-based educational intervention targeting adolescents to mitigate carbonated drink consumption [45] and prevent the resulting nutritional problems that carry into adulthood.

In contrast, the negative association between inadequate FV intake and overweight/obesity found in our study contradicts the widely held view that low consumption of these food groups contributes to excess adiposity among adolescents, which is also highlighted in the existing literature. For example, the Malaysian dataset of the Southeast Asian Nutrition Surveys (SEANUTS Malaysia), which involved 1307 children aged 1–6 years, showed that children who consumed fewer than the recommended two servings per day had significantly higher HAZ values than those who consumed two or more servings daily [46]. Furthermore, the researchers also reported no significant associations between the prevalence of stunting, thinness, or obesity and the achievement of FV intake recommendations. Interestingly, the same study observed that daily energy intake was significantly higher among children who met the FV recommendations after adjusting for sociodemographic variables [46]. This higher energy intake among FV consumers may partly contribute to the negative association observed in our study. Therefore, future studies should objectively measure total energy intake alongside dietary patterns to better elucidate the complex relationship between FV consumption and weight status in this population.

Similarly, a study of school-going adolescents aged 13 to 17 years in Selangor, Malaysia, reported a significantly higher prevalence of adequate fruit intake among adolescents with overweight or obesity compared to those with normal weight or thinness [47]. The observed association may potentially reflect intentional dietary modification, wherein adolescents with a higher BMI increase their intake of fruits and vegetables and physical activity as a weight management strategy or a conscious effort to adopt a healthier lifestyle [48]. This phenomenon is consistent with our previous findings from a nationally representative sample of Malaysian adults, which also demonstrated a significantly higher prevalence of overweight or obesity among individuals who met the adequate daily FV intake recommendations [35]. The reported trend across different age groups within the Malaysian population may be attributed to under-reporting or social desirability bias. It is well-documented in nutritional epidemiology that individuals with a higher BMI are systematically more prone to under-reporting their total energy or ultra-processed food intake [49,50]. Consequently, because our study utilises a cross-sectional design, we cannot preclude reverse causality, where the weight status precedes the dietary and lifestyle changes, a potential explanation for this finding.

A negative association between frequent fast-food consumption and overweight/obesity was observed in the present study, which contradicts with the findings from the published literature [51,52]. The National School-Based Nutrition Survey 2021, conducted among 26,383 Malaysian adolescents aged 10–18 years, found no significant differences in the weekly frequency of fast-food consumption (4–7 days or 1–3 days) between adolescents who were underweight, overweight, or obese compared with those of a normal weight [53]. However, direct comparisons between the overweight/obese groups versus the normal-weight group were not further explored. This discrepancy may be attributable to the study’s definition of fast-food intake, which was limited to food obtained from international fast-food chain restaurants (e.g., Kentucky Fried Chicken, McDonald’s, Domino’s, and Burger King). In Malaysia, adolescents may also frequently consume energy-dense local street foods—such as fried fish crackers, banana fritters (*pisang goreng*), fried tapioca, fried jackfruit, and curry puffs (*karipap*)—that are highly energy-dense, as they are typically deep-fried and rich in fats and carbohydrates, yet they were not captured under the fast-food category. However, these factors were not directly assessed in the present study and should therefore be interpreted cautiously.

The present study showed that insufficient daily dairy intake was associated with lower odds of thinness among Malaysian school-based adolescents, suggesting that adolescents with thinness may be intentionally increasing their dairy consumption as a strategy for weight gain. Furthermore, no association between daily dairy intake and overweight/obesity was observed, which contrasts with previous studies reporting an inverse relationship between total dairy consumption and obesity risk among children and adolescents [54,55]. These conflicting findings may be attributed to the cross-sectional design of the present study, which precludes the establishment of temporal relationships and suggests that the observed dietary patterns may reflect responses to, rather than causes of, the participants’ current nutritional status. Another plausible explanation to the negative associations reported in the present study is the tool used to assess dietary patterns and nutritional outcomes. For instance, the present study utilised the data from AHS 2022 that were collected using the cost-effective GSHS tool. The tool, however, is self-administered (self-report) to collect the health behaviour and protective factor data related to the leading causes of morbidity and mortality among the school-based students [56]. Hence, its reliance on self-report may affect the precise measurement of dietary patterns.

Regarding undernutrition, our study found no significant associations between the measured dietary behaviours and stunting among Malaysian school-based adolescents. Our data aligns with findings by Nachvak and colleagues, who reported no significant link between FV intake and stunting risk among children and early adolescents in Iran [14]. The lack of association in the present study may be attributed to the fact that stunting is a manifestation of chronic, long-term nutritional deficiencies often rooted in the first 1000 days of life. Consequently, the current dietary behaviours captured in this cross-sectional snapshot may not fully reflect the cumulative nutritional history or early-life deprivations that lead to linear growth deficits. Furthermore, while some studies in other LMICs, such as Ethiopia, have linked frequent fast-food intake to higher stunting risk, our null findings suggest that in the Malaysian context, current dietary choices may exert a more detectable influence on immediate weight status (overweight/obesity) than on established height deficits [18].

## 5. Strengths and Limitations

One key strength of the present study is the use of data from the AHS 2022, which is a large and nationally representative sample of Malaysian school-based adolescents. Hence, this ensures that the findings are generalisable to the broader population of Malaysian adolescents attending school. In addition, the AHS 2022 employed the GSHS, a cost-effective standardised questionnaire developed by the WHO and widely used in large-scale international studies involving children and adolescents. The use of this validated instrument allows for meaningful comparisons with findings from other populations and settings. Previous studies have identified socioeconomic status (SES) as a potential confounding factor in the association between frequent fast-food consumption and obesity [52]. In the present study, we accounted for SES, lifestyle factors, and hunger (often considered a proxy for SES) in our detailed regression analyses to address the confounding factors suggested by prior research.

Nevertheless, several limitations should be acknowledged. First, the cross-sectional design of the study precludes the establishment of causal relationships between dietary behaviours and nutritional status among Malaysian school-based adolescents. Second, the GSHS is a self-administered questionnaire, which may be subject to recall bias and social desirability bias. Moreover, the questionnaire includes relatively simple items to assess dietary intake and does not employ comprehensive dietary assessment methods capable of capturing the quantity or portion sizes of food and beverage consumption. These limitations suggest that future studies should adopt prospective study designs and incorporate detailed dietary assessment tools (e.g., semi-quantitative food frequency questionnaire to access dietary behaviours) to obtain quantitative intake data, alongside clinical assessments, to better elucidate causal relationships between dietary behaviours and nutritional outcomes. Furthermore, proximity of a fast-food outlet or grocery store was shown to be associated with an increased risk of overweight among adolescents [57,58]; however, in the present study, proximity was not assessed, further highlighting the importance of considering the built food environment in future research on dietary behaviours and obesity risk. Lifestyle risk factors often exhibit behavioural clustering, which can collectively impact nutritional status. While the secondary dataset utilised here limited our ability to perform a comprehensive analysis of these concurrent factors, it sets the stage for future work. We recommend that future research utilises advanced statistical approaches such as Latent Class Analysis, K-means clustering, or Principal Component Analysis to identify dietary patterns and categorise individuals based on these patterns. This would allow for a more robust exploration of the synergistic effects of lifestyle behaviours on the metabolic health of Malaysian adolescents [59].

## 6. Conclusions

The present study highlighted a high prevalence of unhealthy dietary behaviours and their association with nutritional status among Malaysian school-based adolescents. These findings suggest the importance of food literacy among Malaysian adolescents, which enables and empowers them to make healthier, informed food choices to ensure good nutritional status and build lifelong healthy habits, which is crucial as they gain independence in food selection, impacting physical health and psychological health. Furthermore, it is recommended to promote the Malaysian Healthy Plate (MHP) initiative and reinforce healthy canteen policies in schools. Ensuring the availability and accessibility of balanced, nutrient-rich meals, in line with MHP guidelines, can help improve adolescents’ dietary behaviours and support healthy growth, while also addressing the DBM in this population. Future interventions should also consider integrating family-based nutrition education, coupled with strategies that account for socioeconomic disparities and local food environments, to achieve sustainable improvements in adolescent nutrition.

## Figures and Tables

**Table 1:** Sociodemographic and lifestyle characteristics and nutritional status of Malaysian school-based adolescents aged between 13 and 18 years of age who participated in the AHS 2022 survey (N = 33,523).

	Estimated Population	Count (n)	Prevalence (%)	95% CI
**Sociodemographic**				
Gender				
Male	1,038,709	15,493	50.0	48.0, 52.0
Female	1,029,647	18,030	50.0	48.0, 52.0
Age group (years old)				
≤13–16	1,691,066	27,273	81.4	80.2, 82.6
17–18	386,029	6250	18.6	17.4, 19.8
Ethnicity				
Malay	1,307,669	23,125	63.0	58.2, 67.5
Chinese	376,350	5085	18.1	14.3, 22.7
Indian	123,746	1556	6.0	4.6, 7.6
Bumiputera Sabah and Sarawak	222,937	2963	10.7	9.2, 12.5
Others	46,393	794	2.2	1.8, 2.8
Parental Marital Status				
Married and living together	1,650,157	26,806	79.5	78.5, 80.4
Married but living apart	84,000	1264	4.0	3.7, 4.4
Divorce/widow/separated	302,908	4844	14.6	13.8, 15.4
I do not know	39,239	595	1.9	1.7, 2.2
**Lifestyle factors**				
Non-smoker	1,881,208	30,526	91.0	90.0, 91.8
Non-alcohol drinker	1,908,410	30,526	92.6	91.3, 93.7
**Hunger**				
Always	52,387	869	2.5	2.3, 2.8
Sometimes	605,470	9830	29.2	28.1, 30.3
Never	1,417,740	22,804	68.3	67.1, 69.4
Sedentary behaviour	1,380,667	22,346	66.7	65.3, 68.0
Physically active	443,432	7168	21.4	20.4, 22.4

AHS: Adolescent Health Survey.

## Data Availability

All data generated during this study are included in the published article and its Supplementary Information Files. However, to ensure data protection, the data used in this study are not publicly accessible. They can be obtained from the Sector for Biostatistics and Data Repository, Office of the NIH Manager, National Institutes of Health Malaysia, upon reasonable request and with approval from the Director General of the Ministry of Health Malaysia.

## Supplementary Materials

The following supporting information can be downloaded at: https://www.mdpi.com/article/10.3390/nu18111833/s1, File S1: STROBE Statement—checklist of items that should be included in reports of observational studies; File S2: Table S1: Model diagnostics for complex logistic regression assessing the associations between nutritional status and adequate dietary intake among Malaysian adolescents. Table S2: Prevalence of dietary patterns among Malaysian adolescents. Table S3: Associations between dietary behaviours and nutritional status among Malaysian adolescents. Table S4: Interactions between healthy and unhealthy dietary patterns and their associations with nutritional status among Malaysian adolescents.

## Abbreviations

AHS	Adolescent Health Survey
aORs	Adjusted Odds Ratio
BAZ	BMI for Age Z-score
BMI	Body Mass Index
CI	Confidence Intervals
DBM	Double Burden of Malnutrition
FVs	Fruit and Vegetables
GSHS	Global School-Based Student Health Survey
HAZ	Height-for-Age Z-score
IPH	Institute for Public Health
LMICs	Low- and Middle-income Countries
MHP	Malaysian Healthy Plate
NHMS	National Health and Morbidity Survey
NWO	Normal Weight Obesity
OMR	Optical Mark Recognition
SEANUTS	The Southeast Asian Nutrition Surveys
SES	Socioeconomic Status
STROBE	Strengthening the Reporting of Observational Studies in Epidemiology
WHO	World Health Organization
