# Supplementary material for: Dietary Behaviours and Association with Nutritional Status Among Malaysian School-Based Adolescents: Findings from Adolescent Health Survey 2022
Source: Nutrients. 2026 Jun 5;18(11):1833. doi: 10.3390/nu18111833 (PMC13258971; doi:10.3390/nu18111833)
Supplement: Supplementary file 1 [file nutrients-18-01833-s001.zip › nutrients-4294817-File S2.pdf]

**Table S1**: Model diagnostics for complex logistic regression assessing the associations between nutritional status and adequate dietary intake among Malaysian adolescents.

| Dependent variables | Independent variables | Adjusted OR (95% CI) | P value |
|---|---|---|---|
| Overweight/obese | Fruits and vegetables intake: | | |
| Normal [r] | inadequate | 0.88 (0.81,0.95) | 0.002 |
| | adequate | 1.0 | - |
| | Gender | | |
| | Male | 1.14 (1.05, 1.23) | 0.001 |
| | Female | 1.0 | - |
| | Age | | |
| | ≤ 13-16 | 1.46 (1.37, 1.56) | <0.001 |
| | 17- ≥18 | 1.0 | - |
| | Ethnicity | | |
| | Malay | 1.26 (1.05,1.51) | 0.013 |
| | Chinese | 0.91 (0.74,1.11) | 0.347 |
| | Indian | 1.62 (1.28,2.05) | <0.001 |
| | Bumiputera Sabah & Sarawak | 1.25 (1.01, 1.53) | 0.036 |
| | Others | 1.0 | - |
| | Parental Marital status | | |
| | Married and living together | 0.94 (0.76, 1.16) | 0.559 |
| | Married but living apart | 1.02 (0.79, 1.31) | 0.897 |
| | Divorce/widow/separated | 0.97 (0.77, 1.23) | 0.823 |
| | I do not know | 1.0 | - |
| | Current smoker | | |
| | Yes | 1.09 (0.97, 1.23) | 0.134 |
| | No | 1.0 | - |
| | Current alcohol drinker | | |
| | Yes | 0.88 (0.75, 1.02) | 0.083 |
| | No | 1.0 | - |
| | Hunger | | |

| | | | |
|---|---|---|---|
| | Most of the time/always | 0.57 (0.48, 0.68) | <0.001 |
| | Sometimes/rarely | 0.86 (0.80, 0.92) | <0.001 |
| | Never | 1.0 | - |
| | Physical activity | | |
| | Active | 0.79 (0.73, 0.86) | <0.001 |
| | Inactive | 1.0 | - |
| | Sedentary activities | | |
| | Yes | 1.06 (0.99, 1.13) | 0.099 |
| | No | 1.0 | - |

The model predicted value for nutritional status were 61.1% (AUC under ROC curve: 0.53, 95% CI: 0.52, 0.53, p<0.001). The standard error [highest SE (age): 0.48] and correlation (highest fruit and vegetable intake * hunger: 0.20) are relatively small for the independent variables in the model.

| | | | |
|---|---|---|---|
| Overweight/obese | Fruit intake: | | |
| Normal [r] | | | |
| | inadequate | 0.86 (0.81,0.92) | <0.001 |
| | adequate | 1.0 | - |
| | Gender | | |
| | Male | 1.13 (1.05, 1.22) | <0.001 |
| | Female | 1.0 | - |
| | Age | | |
| | ≤ 13-16 | 1.46 (1.37, 1.56) | <0.001 |
| | 17- ≥18 | 1.0 | - |
| | Ethnicity | | |
| | Malay | 1.26 (1.05,1.51) | 0.015 |
| | Chinese | 0.92 (0.76,1.13) | 0.429 |
| | Indian | 1.62 (1.28,2.06) | <0.001 |
| | Bumiputera Sabah & Sarawak | 1.25 (1.01, 1.53) | 0.037 |
| | Others | 1.0 | - |
| | Parental Marital status | | |
| | Married and living together | 0.94 (0.76, 1.17) | 0.590 |

| | | | |
|---|---|---|---|
| | Married but living apart | 1.03 (0.79, 1.32) | 0.851 |
| | Divorce/widow/separated | 0.98 (0.78, 1.24) | 0.873 |
| | I do not know | 1.0 | - |
| | Current smoker | | |
| | Yes | 1.10 (0.98, 1.24) | 0.121 |
| | No | 1.0 | - |
| | Current alcohol drinker | | |
| | Yes | 0.88 (0.76, 1.02) | 0.086 |
| | No | 1.0 | - |
| | Hunger | | |
| | Most of the time/always | 0.58 (0.48, 0.69) | <0.001 |
| | Sometimes/rarely | 0.86 (0.80, 0.92) | <0.001 |
| | Never | 1.0 | - |
| | Physical activity | | |
| | Active | 0.79 (0.73, 0.86) | <0.001 |
| | Inactive | 1.0 | - |
| | Sedentary activities | | |
| | Yes | 1.06 (0.99, 1.13) | 0.081 |
| | No | 1.0 | - |

The model predicted value for nutritional status were 61.1% (AUC under ROC Curve: 0.56, 95% CI: 0.55, 0.57, p<0.001). The standard error [highest SE (age): 0.21] and correlation (highest fruit intake * hunger: 0.20) are relatively small for the independent variables in the model.

| | | | |
|---|---|---|---|
| Overweight/obese | Vegetable intake: | | |
| Normal [r] | inadequate | | |
| | adequate | 0.91 (0.85, 0.98) | 0.009 |
| | | 1.0 | - |
| | Gender | | |
| | Male | 1.14 (1.06,1.23) | 0.001 |
| | Female | 1.0 | - |
| | Age | | |
| | ≤ 13-16 | 1.46 (1.37,1.56) | <0.001 |
| | 17- ≥18 | 1.0 | - |

| | | |
|---|---|---|
| Ethnicity | | |
| Malay | 1.26 (1.05,1.51) | 0.012 |
| Chinese | 0.90 (0.74,1.11) | 0.904 |
| Indian | 1.62 (1.28,2.05) | <0.001 |
| Bumiputera Sabah & Sarawak | 1.25 (1.02,1.53) | 0.034 |
| Others | 1.0 | - |
| Parental Marital status | | |
| Married and living together | 0.94 (0.76,1.17) | 0.574 |
| Married but living apart | 1.02 (0.79,1.32) | 0.884 |
| Divorce/widow/separated | 0.98 (0.78,1.23) | 0.841 |
| I do not know | 1.0 | - |
| Current smoker | | |
| Yes | 1.10 (0.97,1.23) | 0.127 |
| No | 1.0 | - |
| Current alcohol drinker | | |
| Yes | 0.88 (0.75,1.02) | 0.084 |
| No | 1.0 | - |
| Hunger | | |
| Most of the time/always | 0.57 (0.48,0.68) | <0.001 |
| Sometimes/rarely | 0.85 (0.80,0.91) | <0.001 |
| Never | 1.0 | - |
| Physical activity | | |
| Active | 0.79 (0.73,0.96) | <0.001 |
| Inactive | 1.0 | - |
| Sedentary activities | | |
| Yes | 1.06 (0.99,1.13) | 0.100 |
| No | 1.0 | - |

The model predicted value for nutritional status were 61.1% (AUC under ROC Curve: 0.455, 95% CI: 0.44, 0.45, p<0.001). The standard error [highest SE (age): 0.13] and correlation (highest vegetable intake * current smoker: 0.24) are relatively small for the independent variables in the model.

| | | | |
|---|---|---|---|
| Thinness | Daily Dairy product intake | | |

| | | | |
|---|---|---|---|
| Normal [r] | inadequate | 1.08 (1.00,1.17) | 0.054 |
| | adequate | 1.0 | - |
| | Gender | | |
| | Male | 1.14 (1.06,1.23) | 0.001 |
| | Female | 1.0 | - |
| | Age | | |
| | ≤ 13-16 | 1.46 (1.37,1.56) | <0.001 |
| | 17- ≥18 | 1.0 | - |
| | Ethnicity | | |
| | Malay | 1.26 (1.05,1.51) | 0.014 |
| | Chinese | 0.90 (0.74,1.10) | 0.304 |
| | Indian | 1.63 (1.29,2.06) | <0.001 |
| | Bumiputera Sabah & Sarawak | 1.26 (1.02,1.54) | 0.029 |
| | Others | 1.0 | - |
| | Parental Marital status | | |
| | Married and living together | 0.93 (0.75,1.16) | 0.527 |
| | Married but living apart | 1.01 (1.02,1.54) | 0.029 |
| | Divorce/widow/separated | 0.97 (0.77,1.22) | 0.768 |
| | I do not know | 1.0 | - |
| | Current smoker | | |
| | Yes | 1.09 (0.97,1.23) | 0.146 |
| | No | 1.0 | - |
| | Current alcohol drinker | | |
| | Yes | 0.87 (0.75,1.01) | 0.072 |
| | No | 1.0 | - |
| | Hunger | | |
| | Most of the time/always | 0.57 (0.48,0.68) | <0.001 |
| | Sometimes/rarely | 0.85 (0.80,0.91) | <0.001 |
| | Never | 1.0 | - |
| | Physical activity | | |
| | Active | 0.80 (0.74,0.87) | <0.001 |
| | Inactive | 1.0 | - |

| | | | |
|---|---|---|---|
| | Sedentary activities | | |
| | Yes | 1.05 (0.99,1.12) | 0.124 |
| | No | 1.0 | - |

The model predicted value for nutritional status were 61.2% (AUC under ROC Curve: 0.45, 95% CI: 0.44, 0.45, p<0.001). The standard error [highest SE (age): 0.13] and correlation (highest dairy product intake * physically active: 0.19) are relatively small for the independent variables in the model.

| | | | |
|---|---|---|---|
| Overweight/obese | Fast food intake | | |
| Normal [r] | ≥1day(s)/week | 0.87 (0.82,0.94) | <0.001 |
| | never | 1.0 | - |
| | Gender | | |
| | Male | 1.14(1.06,1.23) | 0.001 |
| | Female | 1.0 | - |
| | Age | | |
| | ≤ 13-16 | 1.46 (1.36,1.56) | <0.001 |
| | 17- ≥18 | 1.0 | - |
| | Ethnicity | | |
| | Malay | 1.26 (1.05,1.51) | 0.014 |
| | Chinese | 0.89 (0.73,1.09) | 0.256 |
| | Indian | 1.62 (1.28,2.05) | 0.039 |
| | Bumiputera Sabah & Sarawak | 1.24 (1.01,1.52) | 0.039 |
| | Others | 1.0 | - |
| | Parental Marital status | | |
| | Married and living together | 0.93 (0.75,1.15) | 0.506 |
| | Married but living apart | 1.00 (0.78,1.29) | 0.983 |
| | Divorce/widow/separated | 0.96 (0.77,1.21) | 0.752 |
| | I do not know | 1.0 | - |
| | Current smoker | | |
| | Yes | 1.09 (0.97,1.23) | 0.157 |
| | No | 1.0 | - |
| | Current alcohol drinker | | |
| | Yes | 0.87 (0.74,1.01) | 0.059 |

| | | | |
|---|---|---|---|
| | No | 1.0 | - |
| | Hunger | | |
| | Most of the time/always | 0.57 (0.48,0.68) | <0.001 |
| | Sometimes/rarely | 0.85 (0.80,0.91) | <0.001 |
| | Never | 1.0 | - |
| | Physical activity | | |
| | Active | 0.80 (0.74,0.87) | <0.001 |
| | Inactive | 1.0 | - |
| | Sedentary activities | | |
| | Yes | 1.05 (0.99,1.13) | 0.126 |
| | No | 1.0 | - |

The model predicted value for nutritional status were 61.1% (AUC under ROC Curve: 0.45, 95% CI: 0.44, 0.45, p=0.003). The standard error [highest SE (age): 0.13] and correlation (highest fast food intake * physically active: -0.20) are relatively small for the independent variables in the model.

| Overweight/obese | Fruit and vegetable intake + fast food intake | | |
|---|---|---|---|
| Normal [r] | IFVI +FF ≥1d(s)/w | 0.99 (0.88,1.12) | 0.880 |
| | AFVI +FF ≥1d(s)/w | 1.08 (0.91,1.27) | 0.393 |
| | IFVI + FF n/w | 0.85 (0.77,0.94) | 0.002 |
| | AFVI + FF n/w | 1.0 | - |
| | Gender | | |
| | Male | 2.46 (2.18,2.78) | <0.001 |
| | Female | 1.0 | - |
| | Age | | |
| | ≤ 13-16 | 0.85 (0.74,0.98) | 0.022 |
| | 17- ≥18 | 1.0 | - |
| | Ethnicity | | |
| | Malay | 1.13 (0.74,1.73) | 0.573 |
| | Chinese | 0.93 (0.58,1.47) | 0.749 |
| | Indian | 2.22 (1.44,3.42) | <0.001 |

| | | |
|---|---|---|
| Bumiputera Sabah& Sarawak | 0.82 (0.53,1.26) | 0.360 |
| Others | 1.0 | - |
| Parental Marital status | | |
| Married and living together | 0.97 (0.69,1.36) | 0.842 |
| Married but living apart | 1.19 (0.80,1.78) | 0.382 |
| Divorce/widow/separated | 0.95 (0.66,1.36) | 0.774 |
| I do not know | 1.0 | - |
| Current smoker | | |
| Yes | 1.30 (1.08,1.56) | 0.005 |
| No | 1.0 | - |
| Current alcohol drinker | | |
| Yes | 1.37 (1.10,1.72) | 0.006 |
| No | 1.0 | - |
| Hunger | | |
| Most of the time/always | 1.19 (0.91,1.55) | 0.210 |
| Sometimes/rarely | 1.14 (1.02,1.27) | 0.024 |
| Never | 1.0 | - |
| Physical activity | | |
| Active | 0.64 (0.54,0.75) | <0.001 |
| Inactive | 1.0 | - |
| Sedentary activities | | |
| Yes | 1.02 (0.91,1.14) | 0.717 |
| No | 1.0 | - |

The model predicted value for nutritional status were 61.2% (AUC under ROC Curve: 0.56, 95% CI: 0.56, 0.57, p<0.001). The standard error [highest SE (age): 0.13] and correlation (highest fruit and vegetable intake + fast food intake * current drinker: -0.16) are relatively small for the independent variables in the model.

| Overweight/obese | Fruit and vegetable intake + carbonated soft drink intake | | |
|---|---|---|---|
| Normal [r] | IFVI +CSD ≥1 t(s)/d | 0.76 (0.67,0.85) | <0.001 |
| | AFVI + CSD ≥1 t(s)/d | 0.80 (0.69,0.93) | 0.004 |
| | IFVI + CSD<1 t(s)/d | 0.800(0.72,0.89) | <0.001 |
| | AFVI + CSD<1d(s)/w | 1.0 | - |

| | | |
|---|---|---|
| Gender | | |
| Male | 1.47 (1.37,1.57) | <0.001 |
| Female | 1.0 | - |
| Age | | |
| ≤ 13-16 | 1.15 (1.06, 1.24) | <0.001 |
| 17- ≥18 | 1.0 | - |
| Ethnicity | | |
| Malay | 1.25 (1.04,1.50) | 0.020 |
| Chinese | 0.90 (0.73,1.10) | 0.256 |
| Indian | 1.61 (1.26,2.04) | <0.001 |
| Bumiputera Sabah & Sarawak | 1.25 (1.02,1.54) | 0.034 |
| Others | 1.0 | - |
| Parental Marital status | | |
| Married and living together | 0.92 (0.74,1.14) | 0.431 |
| Married but living apart | 1.00 (0.77, 1.28) | 0.995 |
| Divorce/widow/separated | 0.96 (0.76,1.21) | 0.718 |
| I do not know | 1.0 | - |
| Current smoker | | |
| Yes | 1.08 (0.96,1.21) | 0.209 |
| No | 1.0 | - |
| Current alcohol drinker | | |
| Yes | 0.87 (0.75,1.01) | 0.064 |
| No | 1.0 | - |
| Hunger | | |
| Most of the time/always | 1.72 (1.44,2.06) | <0.001 |
| Sometimes/rarely | 1.49 (1.25,1.77) | <0.001 |
| Never | 1.0 | - |
| Physical activity | | |
| Active | 0.79 (0.73,0.86) | <0.001 |
| Inactive | 1.0 | - |
| Sedentary activities | | |
| Yes | 1.06 (1.00,1.14) | 0.068 |

| | | |
|---|---|---|
| No | 1.0 | - |

The model predicted value for nutritional status were 61.2% (AUC under ROC Curve: 0.56, 95% CI: 0.56, 0.57, $p<0.001$). The standard error [highest SE (age): 0.13] and correlation (highest fruit and vegetable intake + fast food intake * ethnicity: 0.18) are relatively small for the independent variables in the model.

Abbreviations: AUC: Area under the curve; AFVI: adequate fruit vegetable intake; CI: confident interval; CSD: carbonated soft drink intake; d(s)/w: day(s)/week; FF: fast food intake; IFVI: inadequate fruit vegetable intake; OR: odds ratio; ROC: receiver operating characteristic; n/w: never/week; [r]: reference; t(s)/d: time(s)/day.
**Bold**: Significant level at $p<0.001$ or $p<0.05$ using complex logistic regression.

**Table S2**: Prevalence of dietary patterns among Malaysian adolescents

| Variables | Estimated Population | Count (n) | Prevalence (%) | 95% CI |
|---|---|---|---|---|
| Fruit and vegetable intake | | | | |
| inadequate | 1,742,924 | 28,185 | 83.9 | 83.2, 84.6 |
| adequate | 334,172 | 5,338 | 16.1 | 15.4, 16.8 |
| Fruit intake | | | | |
| inadequate | 1,301,129 | 20,841 | 62.7 | 61.6, 63.8 |
| adequate | 774,014 | 12,655 | 37.3 | 36.2, 38.4 |
| Vegetable intake | | | | |
| inadequate | 1,513,664 | 24,732 | 72.9 | 72.1, 73.8 |
| adequate | 561,456 | 8,762 | 27.1 | 26.2, 27.9 |
| Daily Dairy product intake | | | | |
| inadequate | 1,593,729 | 24,440 | 76.8 | 76.0, 77.6 |
| adequate | 480,850 | 8,047 | 23.2 | 22.4, 24.0 |
| Fast food intake | | | | |
| never/week | 1,340,390 | 21,982 | 64.6 | 63.4, 65.8 |
| ≥ 1 day(s)/week | 734,758 | 11,512 | 35.4 | 34.2, 36.6 |
| Carbonated soft drink intake | | | | |
| < 1 time/day | 1,403,010 | 22,879 | 67.6 | 66.1, 69.1 |
| ≥ 1 time(s)/day | 671,960 | 10,614 | 32.4 | 30.9, 33.9 |
| Fruit and vegetable intake + fast food intake | | | | |
| IFVI + FF ≥1d(s)/w | 627,436 | 9,849 | 30.2 | 29.2, 31.3 |
| AFVI + FF ≥1d(s)/w | 107,321 | 1,663 | 5.2 | 4.8, 5.6 |
| IFVI + FF n/w | 1,113,277 | 18,304 | 53.7 | 52.5, 54.8 |
| AFVI + FF n/w | 226,680 | 3,671 | 10.9 | 10.4, 11.5 |
| Fruit and vegetable intake + carbonated soft drink intake | | | | |
| IFVI +CSD ≥1 t(s)/d | 526,503 | 8,336 | 25.4 | 24.2, 26.6 |
| AFVI + CSD ≥1 t(s)/d | 145,456 | 2,278 | 7.0 | 6.5, 7.5 |
| IFVI + CSD<1 t(s)/d | 1,214,418 | 19,821 | 58.5 | 57.1, 60.0 |
| AFVI + CSD<1d(s)/w | 188,592 | 3,058 | 9.1 | 8.6, 9.6 |
| Daily dairy product intake + fast food intake | | | | |
| ADPI+FF≥1d(s)/w | 135,198 | 2,151 | 6.5 | 6.2, 6.9 |

| | | | | |
|---|---|---|---|---|
| IDPI+FF≥1d(s)/w | 599,428 | 9,357 | 28.9 | 27.8, 30.1 |
| IDPI+FF n/w | 993,946 | 16,076 | 47.9 | 47.0, 48.9 |
| ADPI+FF n/w | 345,346 | 5,891 | 16.7 | 15.9, 17.4 |
| Daily dairy product intake + carbonated soft drink intake | | | | |
| ADPI+CSD≥1 t(s)/d | 222,517 | 3,639 | 10.7 | 10.1, 11.4 |
| IDPI+CSD≥1 t(s)/d | 448,777 | 6,967 | 21.6 | 20.7, 22.7 |
| IDPI+CSD<1 t(s)/d | 1,144,658 | 18,467 | 55.2 | 53.8, 56.6 |
| ADPI+CSD<1 t(s)/d | 258,080 | 4,405 | 12.4 | 11.9, 13.0 |

Abbreviations: AFVI: adequate fruit vegetable intake; ADPI: adequate dairy product intake; CI: confidence interval; CSD: carbonated soft drink intake; d(s)/w: day(s)/week; FF: fast food intake; IDPI: inadequate dairy product intake; IFVI: inadequate fruit vegetable intake: n/w: never/week; t(s)/d: time(s)/day.

**Table S3**: Associations between dietary behaviours and nutritional status among Malaysian adolescents

| Nutritional status (n=33523) | Dietary behaviours (n=33523) | Crude OR (95% CI) | P value | Model 1 [a] | | Model 2 [b] | |
|---|---|---|---|---|---|---|---|
| | | | | OR (95% CI) | P value | OR (95% CI) | P value |
| BAZ | Fruit and vegetable intake | | | | | | |
| Overweight/obese | **inadequate** | **0.8** | **<0.001** | **0.89** | **0.005** | **0.88** | **0.002** |
| | | **(0.80, 0.93)** | | **(0.82,0.97)** | | **(0.81,0.95)** | |
| | adequate | 1.0 | - | 1.0 | - | 1.0 | - |
| Thinness | inadequate | 1.000 | 0.959 | 1.03 | 0.697 | 1.00 | 0.986 |
| | | (0.87, 1.15) | | (0.89,1.18) | | (0.87,1.15) | - |
| | adequate | 1.0 | - | 1.0 | - | 1.0 | |
| Normal | - | 1.0 | - | 1.0 | - | 1.0 | - |
| BAZ | Fruit intake | | | | | | |
| Overweight/obese | **inadequate** | **0.84** | **<0.001** | **0.86** | **<0.001** | **0.86** | **<0.001** |
| | | **(0.78, 0.90)** | | **(0.81,0.92)** | | **(0.81,0.92)** | |
| | adequate | 1.0 | - | 1.0 | - | 1.0 | - |
| Thinness | inadequate | 0.97 | 0.488 | 1.03 | 0.580 | 1.04 | 0.455 |
| | | (0.87,1.07) | | (0.93,1.15) | | (0.93,1.17) | |
| | adequate | 1.0 | - | 1.0 | - | 1.0 | - |
| Normal | - | 1.0 | - | 1.0 | - | 1.0 | - |

| | | | | | | | |
|---|---|---|---|---|---|---|---|
| BAZ | Vegetable intake | | | | | | |
| Overweight/obese | **inadequate** | **0.91** | **0.004** | **0.93** | **0.033** | **0.91** | **0.009** |
| | | **(0.85, 0.97)** | | **(0.87,0.99)** | | **(0.85,0.98)** | |
| | adequate | 1.0 | - | 1.0 | - | 1.0 | - |
| Thinness | inadequate | 1.00 | 0.120 | 1.15 | 0.032 | 1.12 | 0.068 |
| | | (0.98, 1.24) | | (1.01,1.30) | | (0.99,1.26) | - |
| | adequate | 1.0 | - | 1.0 | - | 1.0 | |
| Normal | - | 1.0 | - | 1.0 | - | 1.0 | - |
| BAZ | Dairy product intake | | | | | | |
| Overweight/obese | inadequate | 0.94 | 0.100 | 0.91 | 0.017 | 1.08 | 0.054 |
| | | (0.86,1.01) | | (0.84,0.98) | | (1.00,1.17) | |
| | adequate | 1.0 | - | - | - | 1.0 | - |
| Thinness | **inadequate** | **1.19** | **0.006** | **1.18** | **0.012** | **0.83** | **0.003** |
| | | **(1.05,1.35)** | | **(1.04,1.34)** | | **(0.73,0.94)** | |
| | adequate | 1.0 | - | 1.0 | - | 1.0 | - |
| Normal | - | 1.0 | - | 1.0 | - | 1.0 | - |
| BAZ | Fast food intake | | | | | | |
| Overweight/obese | **≥1day(s)/week** | **0.88** | **<0.001** | **0.88** | **<0.001** | **0.87** | **<0.001** |

| | | | | | | | |
|---|---|---|---|---|---|---|---|
| | | **(0.82,0.94)** | | **(0.82,0.94)** | | **(0.82,0.94)** | |
| | never | 1.0 | - | 1.0 | - | 1.0 | - |
| Thinness | ≥1day(s)/week | 0.99 | 0.910 | 1.02 | 0.750 | 1.02 | 0.751 |
| | | (0.89,1.11) | | (0.91,1.14) | | (0.91,1.14) | |
| | never | 1.0 | - | 1.0 | | 1.0 | |
| Normal | - | 1.0 | - | 1.0 | - | 1.0 | - |

| | | | | | | | |
|---|---|---|---|---|---|---|---|
| BAZ | Carbonated soft drinks intake | | | | | | |
| Overweight/obese | ≥1time(s)/day | 0.94 | 0.087 | 0.89 | 0.002 | 1.11 | 0.004 |
| | | (0.87,1.01) | | (0.83,0.96) | | (1.04,1.20) | |
| | <1time/day[r] | 1.0 | - | 1.0 | | 1.0 | - |
| Thinness | ≥1time(s)/day | 1.07 | 0.238 | 1.07 | 0.289 | 0.96 | 0.472 |
| | | (0.96,1.20) | | (0.95,1.20) | | (0.85,1.08) | |
| | <1time/day | 1.0 | - | 1.0 | | 1.0 | - |
| Normal | - | 1.0 | - | 1.0 | - | 1.0 | - |

| | | | | | | | |
|---|---|---|---|---|---|---|---|
| HAZ | Fruit and vegetable intake | | | | | | |
| Stunting | inadequate | 1.09 | 0.251 | 1.08 | 0.281 | 1.08 | 0.306 |
| | | (0.94,1.26) | | (0.94,1.25) | | (0.93,1.25) | |
| | adequate | 1.0 | - | 1.0 | - | 1.0 | - |
| Normal/tall | - | 1.0 | - | 1.0 | - | 1.0 | - |

| HAZ | Fruit intake | | | | | | |
|---|---|---|---|---|---|---|---|
| Stunting | inadequate | 1.02 | 0.701 | 1.07 | 0.244 | 1.07 | 0.256 |
| | | (0.91,1.15) | | (0.95,1.20) | | (0.95,1.20) | |
| | adequate | - | - | 1.0 | - | 1.0 | - |
| Normal/tall | - | 1.0 | - | 1.0 | - | 1.0 | - |

| HAZ | Vegetable intake | | | | | | |
|---|---|---|---|---|---|---|---|
| Stunting | inadequate | 1.08 | 0.262 | 1.02 | 0.719 | 1.02 | 0.776 |
| | | (0.95,1.22) | | (0.90,1.16) | | (0.90,1.15) | |
| | adequate | - | - | 1.0 | - | 1.0 | - |
| Normal/tall | - | 1.0 | - | 1.0 | - | 1.0 | - |

| HAZ | Fast food intake | | | | | | |
|---|---|---|---|---|---|---|---|
| Stunting | ≥1 day(s)/week | 1.07 | 0.293 | 1.00 | 0.982 | 1.00 | 0.989 |
| | | (0.95,1.20) | | (0.89,1.12) | | (0.89,1.12) | |
| | never | 1.0 | - | 1.0 | - | 1.0 | - |
| Normal/tall | - | 1.0 | - | 1.0 | - | 1.0 | - |

| HAZ | Carbonated soft drink intake | | | | | | |
|---|---|---|---|---|---|---|---|
| Stunting | ≥1time(s)/day | 0.93 | 0.196 | 0.98 | 0.649 | 1.03 | 0.606 |

| | | | | | | | |
|---|---|---|---|---|---|---|---|
| | | (0.83,1.04) | | (0.87,1.09) | | (0.92,1.15) | |
| | <1time/day | 1.0 | - | 1.0 | - | 1.0 | - |
| Normal/tall | - | 1.0 | - | 1.0 | - | 1.0 | - |

Complex logistic regression was performed.
[a] Model 1: adjusted by age, sex, race, marital status, smoker and alcohol drinker.
[b] Model 2: adjusted by model 1, hunger status, physical activity and sedentary activity.
Abbreviations: BAZ: BMI for age z-score; CI: confident interval; HAZ: Height for age z-score; OR: odds ratio.

**Table S4**: Interactions between healthy and unhealthy dietary patterns and their associations with nutritional status among Malaysian adolescents

| Depedent variables | Indepedent variables | Crude OR (95% CI) | P value | Model 1 [a] | | Model 2 [b] | |
|---|---|---|---|---|---|---|---|
| | | | | OR (95% CI) | P value | OR (95% CI) | P value |
| BAZ | Fruit and vegetable intake + fast food intake | | | | | | |
| Overweight/obese | IFVI + FF ≥1d(s)/w | 0.97 (0.86, 1.09) | 0.589 | 0.98 (0.87,1.10) | 0.729 | 0.99 (0.88,1.12) | 0.880 |
| | AFVI + FF ≥1d(s)/w | 1.07 (0.90,1.09) | 0.448 | 1.05 (0.89,1.24) | 0.539 | 1.08 (0.91,1.27) | 0.393 |
| | IFVI + FF n/w | **0.84 (0.76, 0.92)** | **<0.001** | **0.85 (0.77,0.94)** | **0.002** | **0.85 (0.77,0.94)** | **0.002** |
| | AFVI + FF n/w | 1.0 | - | 1.0 | - | 1.0 | - |
| Thinness | IFVI + FF ≥1d(s)/w | 1.00 (0.83,1.21) | 0.981 | 1.000 (0.82,1.20) | 0.913 | 0.95 (0.79,1.15) | 0.625 |
| | AFVI + | 0.98 | 0.904 | 0.92 | 0.594 | 0.92 | 0.615 |

| | | | | | | | |
|---|---|---|---|---|---|---|---|
| | FF ≥1d(s)/w | (0.83,1.21) | | (0.67,1.25) | | (0.68,1.26) | |
| | IFVI < | 0.99 | 0.871 | 1.02 | | 0.98 | 0.843 |
| | 5t(s)/d+ | (0.82, 1.18) | | (0.86,1.21) | 0.837 | (0.86,1.17) | |
| | FF n/w | | | | | | |
| | | 1.0 | - | 1.0 | - | 1.0 | - |
| | AFVI + | | | | | | |
| | FF n/w | | | | | | |
| Normal | - | 1.0 | - | 1.0 | - | 1.0 | - |
| BAZ | Fruit and vegetable intake + carbonated soft drink intake | | | | | | |
| Overweight/obese | IFVI + | **0.77** | **<0.001** | **0.77** | **<0.001** | **0.76** | **<0.001** |
| | CSD ≥1 t(s)/d | **(0.68,0.86)** | | **(0.68,0.86)** | | **(0.67,0.85)** | |
| | AFVI + | **0.82** | **0.009** | **0.80** | **0.003** | **0.80** | **0.004** |
| | CSD ≥1 t(s)/d | **(0.70,0.95)** | | **(0.69,0.93)** | | **(0.69,0.93)** | |
| | IFVI + | **0.80** | **<0.001** | **0.82** | **<0.001** | **0.80** | **<0.001** |
| | CSD<1 t(s)/d | **(0.72,0.89)** | | **(0.74,0.91)** | | **(0.72,0.89)** | |

| | | | | | | | |
|---|---|---|---|---|---|---|---|
| | AFVI + CSD<1d(s)/w | 1.0 | - | 1.0 | - | 1.0 | - |
| Thinness | IFVI + CSD ≥1 t(s)/d | 1.13 (0.93,1.36) | 0.225 | 1.17 (0.96,1.42) | 0.131 | 1.09 (0.89,1.34) | 0.404 |
| | AFVI + CSD ≥1 t(s)/d | 1.22 (0.95,1.57) | 0.125 | 1.20 (0.92,1.56) | 0.174 | 1.16 (0.88,1.52) | 0.286 |
| | IFVI + CSD<1 t(s)/d | 1.08 (0.90,1.30) | 0.402 | 1.11 (0.92,1.34) | 0.258 | 1.06 (0.88,1.28) | 0.528 |
| | AFVI + CSD<1d(s)/w | 1.0 | - | 1.0 | - | 1.0 | - |
| Normal | - | 1.0 | - | 1.0 | - | 1.0 | - |
| BAZ | Dairy product intake + fast food intake | | | | | | |
| Overweight/obese | ADPI+ FF≥1d(s)/w | 1.08 (0.95,1.24) | 0.249 | 1.09 (0.95,1.25) | 0.246 | 1.09 (0.95,1.26) | 0.198 |

| | | | | | | | |
|---|---|---|---|---|---|---|---|
| | IDPI+ FF≥1d(s)/w | **1.19 (1.07,1.32)** | **0.001** | **1.23 (1.11,1.36)** | **<0.001** | **1.21 (1.09,1.34)** | **<0.001** |
| | IDPI + FF n/w | 1.04 (0.95,1.14) | 0.423 | 1.07 (0.97,1.17) | 0.158 | 1.05 (0.96,1.15) | 0.289 |
| | ADPI + FF n/w [r] | 1.0 | - | 1.0 | - | 1.0 | - |
| Thinness | ADPI+ FF≥1d(s)/w | 1.03 (0.82,1.31) | 0.785 | 1.00 (0.79,1.26) | 0.995 | 1.00 (0.79,1.27) | 0.986 |
| | IDPI+ FF≥1d(s)/w | 0.86 (0.73,1.01) | 0.068 | 0.85 (0.72,1.00) | 0.847 | **0.83 (0.70,0.98)** | **0.025** |
| | IDPI + FF n/w | 0.84 (0.72,0.99) | 0.038 | 0.85 (0.73,1.00) | 0.853 | **0.83 (0.71,0.98)** | **0.026** |
| | ADPI + FF n/w | 1.0 | - | 1.0 | - | 1.0 | - |

| | | | | | | | |
|---|---|---|---|---|---|---|---|
| Normal | - | 1.0 | - | 1.0 | - | 1.0 | - |
| BAZ | Daily dairy product intake + carbonated soft drink intake | | | | | | |
| Overweight/obese | ADPI+ CSD≥1 t(s)/d | 0.91 (0.80,1.03) | 0.144 | **0.86 (0.76,0.97)** | **0.013** | **0.85 (0.76,0.97)** | **0.011** |
| | IDPI+ CSD≥1 t(s)/d | 0.99 (0.88,1.12) | 0.923 | 0.97 (0.86,1.09) | 0.559 | 0.95 (0.84,1.07) | 0.407 |
| | IDPI+ CSD<1 t(s)/d | 1.04 (0.93,1.15) | 0.497 | 1.05 (0.95,1.17) | 0.335 | 1.03 (0.93,1.14) | 0.609 |
| | ADPI+ CSD<1 t(s)/d | 1.0 | - | 1.0 | - | 1.0 | - |
| Thinness | ADPI+ CSD≥1 t(s)/d | 1.08 (0.90,1.29) | 0.390 | 1.05 (0.88,1.26) | 0.608 | 1.01 (0.84,1.22) | 0.890 |
| | IDPI+ CSD≥1 t(s)/d | 0.89 (0.74,1.08) | 0.225 | 0.89 (0.74,1.08) | 0.254 | 0.84 (0.69,1.02) | 0.085 |

| | | | | | | | |
|---|---|---|---|---|---|---|---|
| | IDPI+ CSD<1 t(s)/d | 0.87 (0.74,1.02) | 0.082 | 0.86 (0.73,1.02) | 0.075 | 0.83 (0.71,0.98) | 0.026 |
| | ADPI+ CSD<1 t(s)/d | 1.0 | - | 1.0 | - | 1.0 | - |
| Normal | - | 1.0 | - | 1.0 | - | 1.0 | - |
| HAZ | Fruit and vegetable intake + fast food intake | | | | | | |
| Stunting | IFVI + FF ≥1d(s)/w | 1.01 (0.83,1.23) | 0.907 | 1.07 (0.88,1.30) | 0.487 | 1.02 (0.85,1.23) | 0.848 |
| | AFVI + FF ≥1d(s)/w | 0.90 (0.63,1.23) | 0.572 | 0.96 (0.67,1.38) | 0.822 | 1.08 (0.80,1.46) | 0.599 |
| | IFVI + FF n/w | 1.07 (0.91,1.28) | 0.408 | 1.07 (0.90,1.27) | 0.473 | 0.96 (0.81,1.14) | 0.615 |
| | AFVI + FF n/w | 1.0 | - | 1.0 | - | 1.0 | - |

| | | | | | | | |
|---|---|---|---|---|---|---|---|
| Normal/tall | - | 1.0 | - | 1.0 | - | 1.0 | - |
| HAZ | Fruit and vegetable intake + carbonated soft drink intake | | | | | | |
| Stunting | IFVI + CSD ≥1 t(s)/d | 1.18 (0.95,1.47) | 0.138 | 1.18 (0.95,1.48) | 0.140 | 1.17 (0.94,1.47) | 0.166 |
| | AFVI + CSD ≥1 t(s)/d | 1.05 (0.82,1.36) | 0.690 | 1.14 (0.88,1.47) | 0.329 | 1.12 (0.87,1.46) | 0.374 |
| | IFVI + CSD<1 t(s)/d | 1.08 (0.89,1.31) | 0.419 | 1.04 (0.86,1.26) | 0.684 | 1.04 (0.85,1.25) | 0.726 |
| | AFVI + CSD<1d(s)/w | 1.0 | - | 1.0 | - | 1.0 | - |
| Normal/tall | - | 1.0 | - | 1.0 | - | 1.0 | - |
| HAZ | Dairy product intake + fast food intake | | | | | | |
| Stunting | ADPI+ FF≥1d(s)/w | 0.92 (0.73,1.17) | 0.501 | 0.97 (0.76,1.24) | 0.822 | 1.03 (0.82,1.29) | 0.804 |
| | IDPI+ | 0.96 | 0.663 | 1.05 | 0.532 | 1.29 | 0.002 |

| | | | | | | | |
|---|---|---|---|---|---|---|---|
| | FF≥1d(s)/w | (0.81,1.14) | | (0.89,1.24) | | (1.10,1.52) | |
| | IDPI + FF n/w | 1.03 (0.88,1.19) | 0.740 | 1.05 (0.90,1.23) | 0.529 | 1.23 (1.05,1.44) | 0.013 |
| | ADPI + FF n/w | 1.0 | - | 1.0 | - | 1.0 | - |
| Normal/tall | - | 1.0 | - | 1.0 | - | 1.0 | - |
| HAZ | Dairy product intake + carbonated soft drink intake | | | | | | |
| Stunting | ADPI+ CSD≥1 t(s)/d | 1.11 (0.88,1.38) | 0.378 | 1.10 (0.88,1.37) | 0.414 | 1.10 (0.88,1.37) | 0.423 |
| | IDPI+ CSD≥1 t(s)/d | 1.14 (0.93,1.38) | 0.202 | 1.12 (0.92,1.36) | 0.250 | 1.10 (0.91,1.34) | 0.333 |
| | IDPI+ CSD<1 t(s)/d | 1.05 (0.88,1.26) | 0.581 | 1.10 (0.92,1.32) | 0.287 | 1.08 (0.90,1.30) | 0.398 |
| | ADPI+ | 1.0 | - | 1.0 | - | 1.0 | - |

| | | | | | | | |
|---|---|---|---|---|---|---|---|
| | CSD<1 t(s)/d | | | | | | |
| Normal/tall | - | 1.0 | - | 1.0 | - | 1.0 | - |

Complex logistic regression was performed.

[a] Model 1: adjusted by age, sex, race, marital status, smoker and alcohol drinker.

[b] Model 2: adjusted by model 1, hunger status, physical activity and sedentary activity.

**Bold**: Significant level at $p<0.001$ or $p<0.05$ using complex logistic regression.

Abbreviations: AFVI: adequate fruit vegetable intake; ADPI: adequate dairy product intake; BAZ: BMI for age z-score; CI: confident interval; CSD: carbonated soft drink intake; d(s)/w: day(s)/week; FF: fast food intake; HAZ: height for age z-score; IDPI: inadequate dairy product intake; IFVI: inadequate fruit vegetable intake: OR: odds ratio; n/w: never/week; t(s)/d: time(s)/day.
