# Supplementary material for: Dietary Behaviours and Association with Nutritional Status Among Malaysian School-Based Adolescents: Findings from Adolescent Health Survey 2022
Source: Nutrients. 2026 Jun 5;18(11):1833. doi: 10.3390/nu18111833 (PMC13258971; doi:10.3390/nu18111833)
Supplement: Supplementary file 1 [file nutrients-18-01833-s001.zip › nutrients-4294817-File S1_STROBE-checklist.pdf]

STROBE Statement—checklist of items that should be included in reports of observational studies

| | Item No. | Recommendation | Page No. | Relevant text from manuscript |
|---|---|---|---|---|
| **Title and abstract** | 1 | (*a*) Indicate the study's design with a commonly used term in the title or the abstract | 1 | Dietary Behaviours and Association with Nutritional Status among Malaysian School-Based Adolescents: Findings from Adolescent Health Survey 2022 |
| | | (*b*) Provide in the abstract an informative and balanced summary of what was done and what was found | 1 | This study determined the prevalence of dietary behaviours and examined their relationship with nutritional status among Malaysian school-based adolescents. Data from 33,523 adolescents who participated in the Adolescent Health Survey (AHS 2022) were analysed. Multiple logistic regression was employed to assess associations between dietary behaviours and nutritional status, adjusting for potential confounders. Our findings demonstrated a double burden of malnutrition, with 6.8% stunting, 8.3% thinness, and 30.5% overweight/obese. High prevalence of inadequate daily intake of fruit and vegetables (FV) (83.9%) and insufficient daily dairy consumption (62.7%) was observed. Approximately one-third of adolescents reported frequent fast-food consumption (i.e. at least one day per week) (35.4%) and daily intake of carbonated soft drinks (32.4%). Daily carbonated soft drink consumption was associated with higher odds of overweight/obesity (aOR:1.11; 95% CI:1.04–1.20), highlighting the need to prioritize the public health strategies aimed at reducing sugar intake. Conversely, inadequate FV intake (aOR:0.88; 95% CI:0.81–0.95) and frequent fast-food consumption (aOR:0.87; 95% CI:0.82–0.94) were associated with lower odds of overweight/obesity, while insufficient daily dairy intake was associated with lower odds of thinness (aOR:0.83; 95% CI:0.73–0.94). These negative associations should be interpreted cautiously due to possible reverse causality and reporting bias. The findings highlight the importance of strengthening nutrition education and food environment among Malaysian adolescents. |
| | | **Introduction** | | |
| Background/rationale | 2 | Explain the scientific background and rationale for the investigation being reported | 1-3 | a) Adolescence is a critical developmental stage characterized by rapid physical, psychological, and social changes, with nutritional requirements increasing substantially during this period.<br>b) Adolescents represent approximately 1.8 billion people globally, with the majority residing in low- and middle-income countries, where the double burden of malnutrition (DBM) is increasingly prevalent. |

| | | | | |
|---|---|---|---|---|
| | | | | c) Global evidence indicates coexistence of undernutrition (thinness and stunting) and overnutrition (overweight and obesity) among adolescents, highlighting an important public health concern.<br>d) Unhealthy dietary behaviours, including inadequate fruit and vegetable intake, frequent fast-food consumption, and high intake of sugar-sweetened beverages, contribute to poor nutritional outcomes among adolescents worldwide.<br>e) Previous studies examining associations between dietary behaviours and nutritional status have reported inconsistent findings across populations, suggesting the need for further investigation in diverse settings.<br>f) Emerging evidence also suggests that dietary behaviours interact with other lifestyle factors, such as sleep and meal timing, influencing body composition and metabolic health outcomes.<br>g) Malaysia is experiencing a growing double burden of malnutrition among adolescents, accompanied by unhealthy dietary practices, including low fruit and vegetable intake and high consumption of carbonated beverages and fast foods.<br>h) Despite increasing concern regarding adolescent nutrition in Malaysia, limited evidence exists on the association between dietary behaviours and nutritional status among Malaysian adolescents. |
| Objectives | 3 | State specific objectives, including any prespecified hypotheses | 3 | a) Therefore, this study was conducted to describe dietary behaviours and nutritional status among Malaysian adolescents and to examine the associations between dietary behaviours and nutritional outcomes using data from the AHS 2022.<br>b) Prespecified hypothesis: unhealthy dietary behaviours (e.g., inadequate fruit and vegetable intake, frequent fast-food consumption, and frequent carbonated soft drink intake) would be associated with poorer nutritional outcomes, including thinness, overweight/obesity, and stunting among Malaysian adolescents. |
| | | **Methods** | | |
| Study design | 4 | Present key elements of study design early in the paper | 3-4 | a) The study utilised secondary data from the Adolescent Health Survey (AHS) 2022.<br>b) The AHS 2022 was a nationwide cross-sectional study conducted by the Ministry of Health Malaysia in collaboration with the Ministry of Education Malaysia.<br>c) The survey aimed to provide nationally representative data on health behaviours and related factors among Malaysian school-based adolescents aged 13 to 18 years.<br>d) A two-stage stratified cluster sampling design was used to obtain a nationally representative |

| | | | | |
|---|---|---|---|---|
| | | | | sample of Malaysian secondary school students.<br>e) The present study performed secondary analysis of the AHS 2022 data stored in the NIH-Data Repository System, with ethical approval obtained from the Medical Research and Ethics Committee (MREC), Ministry of Health Malaysia.<br>f) The study was prepared in accordance with the STROBE guideline for cross-sectional studies. |
| Setting | 5 | Describe the setting, locations, and relevant dates, including periods of recruitment, exposure, follow-up, and data collection | 4 | The study was conducted among Malaysian school-based adolescents aged 13–18 years.<br>a) The survey was implemented across all 13 Malaysian states and three Federal Territories (Kuala Lumpur, Labuan, and Putrajaya).<br>b) Data collection was conducted between June and July 2022.<br>c) Data were collected during school hours using supervised optical mark recognition (OMR) questionnaires.<br>d) As this was a cross-sectional study, no follow-up period was applicable. |
| Participants | 6 | (*a*) *Cohort study*—Give the eligibility criteria, and the sources and methods of selection of participants. Describe methods of follow-up<br>*Case-control study*—Give the eligibility criteria, and the sources and methods of case ascertainment and control selection. Give the rationale for the choice of cases and controls<br>*Cross-sectional study*—Give the eligibility criteria, and the sources and methods of selection of participants | 4-5 | The study included Malaysian school-based adolescents aged 13–18 years who participated in the Adolescent Health Survey (AHS) 2022. Participants were selected using a two-stage stratified cluster sampling design involving probability proportional to size sampling of secondary schools followed by systematic selection of classes within schools. All students in selected classes were invited to participate. Participants with missing data on key study variables were excluded from the final analysis. |
| | | (*b*) *Cohort study*—For matched studies, give matching criteria and number of exposed and unexposed<br>*Case-control study*—For matched studies, give matching criteria and the number of controls per case | | |
| Variables | 7 | Clearly define all outcomes, exposures, | 4-5 | **<u>Outcomes</u>** |

| | |
|---|---|
| predictors, potential confounders, and effect modifiers. Give diagnostic criteria, if applicable | a) The primary outcome variables were nutritional status indicators, namely body mass index-for-age z-score (BAZ) and height-for-age z-score (HAZ), calculated according to the World Health Organization (WHO) 2007 Growth Reference for adolescents aged 5–19 years.<br>b) Nutritional status was categorised as thinness, normal weight, overweight, obesity, and stunting based on WHO-recommended cut-off values. Thinness was defined as BAZ < −2 SD, overweight as BAZ > +1 SD, obesity as BAZ > +2 SD, and stunting as HAZ < −2 SD.<br><br>**<u>Exposures / Independent Variables</u>**<br>c) The exposure variables were dietary behaviours, comprising both healthy and unhealthy dietary indicators.<br>d) Healthy dietary behaviours included fruit and vegetable (FV) intake and daily dairy product intake. Adequate FV intake was defined as consuming at least two servings of fruit and at least three servings of vegetables daily. Adequate dairy intake was defined as consuming dairy products at least twice daily.<br>e) Unhealthy dietary behaviours included fast-food consumption and carbonated soft drink intake. Frequent fast-food intake was defined as consumption on one or more days per week, while frequent carbonated soft drink intake was defined as consumption at least once per day.<br>f) Combined dietary behaviour variables were additionally created to examine the joint effects of healthy and unhealthy dietary behaviours on nutritional status.<br><br>**<u>Potential Confounders / Predictors</u>**<br>g) Potential confounding variables included socio-demographic characteristics and lifestyle-related factors.<br>h) Socio-demographic variables included sex, age group, ethnicity, and parental marital status.<br>i) Lifestyle-related variables included smoking status, alcohol consumption, physical activity, sedentary behaviour, and self-reported hunger status as a proxy indicator of food insecurity.<br>j) Physical activity was classified according to WHO recommendations, where adolescents performing at least 60 minutes of moderate-to-vigorous physical activity on five or more days per week were considered physically active. Sedentary behaviour was defined as spending three or more hours per day in sitting or screen-based leisure activities outside schoolwork.<br><br>**<u>Effect Modifiers</u>** |

| | | | | |
|---|---|---|---|---|
| | | | | k) Combined dietary behaviour variables and exploratory two-way interaction analyses were used to assess potential interaction effects between healthy and unhealthy dietary behaviours on nutritional status outcomes. |
| Data sources/ measurement | 8* | For each variable of interest, give sources of data and details of methods of assessment (measurement). Describe comparability of assessment methods if there is more than one group | 4 | All participants completed the same standardised OMR questionnaire, and data collection procedures were standardised through centralised training and supervision to ensure consistency and comparability across study sites. |
| Bias | 9 | Describe any efforts to address potential sources of bias | 4 | a) A nationwide two-stage stratified cluster sampling design with sampling weights was used to minimise selection bias.<br>b) Standardised training and supervised data collection procedures were implemented to reduce measurement bias.<br>c) Quality control procedures, including field verification and data checking for completeness and consistency, were conducted to minimise information bias.<br>f) Participants with missing data on key variables were excluded using a complete-case approach. |
| Study size | 10 | Explain how the study size was arrived at | 4 | a) The sample size was determined using a single-proportion formula based on prevalence estimates from the previous Adolescent Health Survey (AHS).<br>b) The calculation applied a 95% confidence level, margin of error between 1% and 5%, and a design effect of 2 to account for cluster sampling.<br>c) The estimated sample size was increased by 20% to account for potential non-response.<br>d) The final sample comprised 33,523 Malaysian school-based adolescents aged 13–18 years. |

Continued on next page

| | | | | |
|---|---|---|---|---|
| Quantitative variables | 11 | Explain how quantitative variables were handled in the analyses. If applicable, describe which groupings were chosen and why | 4 | a) Nutritional status variables were converted into categorical variables using WHO 2007 Growth Reference cut-offs for BAZ and HAZ.<br>b) Dietary behaviour variables were categorised based on recommended intake thresholds and frequency of consumption.<br>c) Age was grouped into 13–16 years and 17–18 years to reflect the Malaysian secondary school system structure.<br>d) Physical activity was categorised according to WHO recommendations, where adolescents performing at least 60 minutes of moderate-to-vigorous physical activity on five or more days per week were classified as physically active.<br>e) Sedentary behaviour was categorised as spending three or more hours per day in sitting or screen-based leisure activities outside schoolwork.<br>f) Logistic regression analyses were conducted using dichotomised nutritional status outcomes and binary dietary behaviour variables. |
| Statistical methods | 12 | (*a*) Describe all statistical methods, including those used to control for confounding | 5-6 | a) All analyses were performed using IBM SPSS Statistics version 31 with Complex Samples procedures to account for the two-stage stratified cluster sampling design and sampling weights.<br>b) Descriptive statistics were used to summarise socio-demographic characteristics, lifestyle factors, dietary behaviours, and nutritional status.<br>c) Categorical variables were presented as weighted percentages with 95% confidence intervals (CI).<br>d) Associations between dietary behaviours and nutritional status were analysed using complex logistic regression analyses.<br>e) Multivariable models adjusted for potential confounders, including age, sex, ethnicity, parental marital status, smoking status, alcohol consumption, hunger status, physical activity, and sedentary behaviour.<br>f) Adjusted odds ratios (aOR) and 95% confidence intervals were reported, with statistical significance set at $p < 0.05$. |
| | | (*b*) Describe any methods used to examine subgroups and interactions | 5-6 | a) Combined dietary behaviour variables were created to examine the joint effects of healthy and unhealthy dietary behaviours on nutritional status outcomes.<br>b) Exploratory two-way interaction analyses were conducted for combined dietary behaviour variables, including fruit and vegetable intake with fast-food intake, fruit and vegetable intake with carbonated soft drink intake, dairy intake with fast-food intake, and dairy intake with carbonated soft drink intake. |

| | | | | |
|---|---|---|---|---|
| | | | | c) The healthiest dietary behaviour category was used as the reference group in interaction analyses. |
| | | (*c*) Explain how missing data were addressed | 5-6 | a) Participants with missing data on key variables, including dietary behaviours, anthropometric outcomes, or covariates used in regression models, were excluded using a complete-case approach.<br>b) The proportion of missing data was low and considered unlikely to introduce significant bias; therefore, no imputation methods were performed. |
| | | (*d*) *Cohort study*—If applicable, explain how loss to follow-up was addressed<br>*Case-control study*—If applicable, explain how matching of cases and controls was addressed<br>*Cross-sectional study*—If applicable, describe analytical methods taking account of sampling strategy | 5-6 | a) All analyses were performed using IBM SPSS Statistics version 31 Complex Samples procedures to account for the two-stage stratified cluster sampling design.<br>b) Sampling weights were applied to ensure nationally representative estimates.<br>c) Complex logistic regression analyses were conducted to account for clustering and stratification in the sampling design. |
| | | (*e*) Describe any sensitivity analyses | 6 | a) Model predictive performance was evaluated using receiver operating characteristic (ROC) curve analysis and classification tables.<br>b) Variance inflation factor analysis was performed to assess multicollinearity among independent variables.<br>c) The progressive inclusion of covariates from Model 1 to Model 2 resulted in minimal changes in effect estimates, supporting model stability and robustness of the findings. |
| **Results** | | | | |
| Participants | 13* | (a) Report numbers of individuals at each stage of study—eg numbers potentially eligible, examined for eligibility, confirmed eligible, included in the study, completing follow-up, and analysed | 4 | a) A total of approximately 36,000 adolescents were initially targeted in the AHS 2022 sampling process.<br>b) The final analysed sample comprised 33,523 Malaysian school-based adolescents aged 13–18 years.<br>c) Participants with missing data on key variables, including dietary behaviours, anthropometric outcomes, or covariates, were excluded from the analysis using a complete-case approach.<br>d) As this was a cross-sectional study, no follow-up stage was applicable. |
| | | (b) Give reasons for non-participation at each stage | 4,7 | a) The manuscript states that the estimated sample size was increased by 20% to account for potential non-response.<br>b) Participants with missing data on key variables were excluded from the final analysis using a |

| | | | | |
|---|---|---|---|---|
| | | | | complete-case approach.<br>c) Specific reasons for non-participation or exclusion at each stage were not further described in the manuscript. |
| | | (c) Consider use of a flow diagram | - | No participant flow diagram was included in the present manuscript. |
| Descriptive data | 14* | (a) Give characteristics of study participants (eg demographic, clinical, social) and information on exposures and potential confounders | 8-10 | a) The study included 33,523 Malaysian school-based adolescents aged 13–18 years.<br>b) Most participants were aged 13–16 years (81.4%), Malay (63.0%), and had parents who were married and living together (79.5%).<br>c) Half of the participants were male (50.0%).<br>d) Most adolescents were non-smokers (91.0%) and non-alcohol drinkers (92.6%).<br>e) Approximately 66.7% reported sedentary behaviour, while 21.4% were physically active.<br>f) Exposure variables included fruit and vegetable intake, dairy intake, fast-food consumption, and carbonated soft drink intake.<br>g) Potential confounders included socio-demographic and lifestyle-related variables. |
| | | (b) Indicate number of participants with missing data for each variable of interest | 6 | The manuscript stated that the proportion of missing data was low; however, the exact number of missing values for each variable was not separately reported. |
| | | (c) *Cohort study*—Summarise follow-up time (eg, average and total amount) | | |
| Outcome data | 15* | *Cohort study*—Report numbers of outcome events or summary measures over time | | |
| | | *Case-control study*—Report numbers in each exposure category, or summary measures of exposure | | |
| | | *Cross-sectional study*—Report numbers of outcome events or summary measures | 8-10 | a) Among Malaysian school-based adolescents, 61.2% had normal BMI-for-age z-scores (BAZ), 30.5% were overweight/obese, and 8.3% were classified as thin.<br>b) Based on height-for-age z-scores (HAZ), 6.8% of adolescents were stunted.<br>c) The prevalence of inadequate fruit and vegetable intake was 83.9%, while 76.8% reported insufficient daily dairy intake.<br>d) Approximately 64.6% of adolescents consumed fast food at least once per week, and 67.6% reported daily carbonated soft drink intake. |
| Main results | 16 | (*a*) Give unadjusted estimates and, if applicable, confounder-adjusted estimates and | 11 | a) Complex logistic regression analyses were performed to estimate associations between dietary behaviours and nutritional status. |

| | | | |
|---|---|---|---|
| | their precision (eg, 95% confidence interval). Make clear which confounders were adjusted for and why they were included | | b) Adjusted odds ratios (aOR) with 95% confidence intervals (CI) were reported.<br>c) Daily carbonated soft drink intake was associated with higher odds of overweight/obesity (aOR: 1.11; 95% CI: 1.04–1.20).<br>d) Inadequate fruit and vegetable intake was associated with lower odds of overweight/obesity (aOR: 0.88; 95% CI: 0.81–0.95).<br>e) Frequent fast-food consumption was associated with lower odds of overweight/obesity (aOR: 0.87; 95% CI: 0.82–0.94).<br>f) Insufficient daily dairy intake was associated with lower odds of thinness (aOR: 0.83; 95% CI: 0.73–0.94).<br>g) The regression models adjusted for socio-demographic and lifestyle-related confounders, including age, sex, ethnicity, parental marital status, smoking status, alcohol consumption, hunger status, physical activity, and sedentary behaviour, as these factors may influence adolescent nutritional status outcomes. |
| | (*b*) Report category boundaries when continuous variables were categorized | 5-6 | a) BMI-for-age z-score (BAZ) was categorised as thinness (< −2 SD), normal (≥ −2 SD to < +1 SD), overweight (> +1 SD), and obesity (> +2 SD) based on WHO 2007 Growth Reference cut-offs.<br>b) Height-for-age z-score (HAZ) was categorised as stunted (< −2 SD) or normal/tall (≥ −2 SD).<br>c) Fruit intake was categorised as adequate (≥ 2 servings/day) or inadequate (< 2 servings/day), while vegetable intake was categorised as adequate (≥ 3 servings/day) or inadequate (< 3 servings/day).<br>d) Daily dairy intake was categorised as adequate (≥ 2 times/day) or inadequate (< 2 times/day).<br>e) Fast-food intake was categorised as frequent (≥ 1 day/week) or non-frequent (< 1 day/week).<br>f) Carbonated soft drink intake was categorised as daily (≥ 1 time/day) or non-daily (< 1 time/day).<br>g) Age was grouped into 13–16 years and 17–18 years. |
| | (*c*) If relevant, consider translating estimates of relative risk into absolute risk for a meaningful time period | | Not applicable |

Continued on next page

| | | | | |
|---|---|---|---|---|
| Other analyses | 17 | Report other analyses done—eg analyses of subgroups and interactions, and sensitivity analyses | 11-13 | a) Combined dietary behaviour analyses were conducted to examine the joint effects of healthy and unhealthy dietary behaviours on nutritional status outcomes.<br>b) Exploratory two-way interaction analyses were performed for combinations of fruit and vegetable intake, dairy intake, fast-food consumption, and carbonated soft drink intake.<br>c) Sensitivity analyses included receiver operating characteristic (ROC) curve analysis, classification tables, and multicollinearity assessment to evaluate model performance and stability.<br>d) The progressive inclusion of covariates from Model 1 to Model 2 resulted in minimal changes in effect estimates, supporting robustness of the findings. |
| **Discussion** | | | | |
| Key results | 18 | Summarise key results with reference to study objectives | 11-12 | a) The study identified a double burden of malnutrition among Malaysian school-based adolescents, with 30.5% overweight/obesity, 8.3% thinness, and 6.8% stunting.<br>b) High prevalence of unhealthy dietary behaviours was observed, including inadequate fruit and vegetable intake, insufficient dairy intake, frequent fast-food consumption, and daily carbonated soft drink intake.<br>c) Daily carbonated soft drink intake was associated with higher odds of overweight/obesity, while inadequate fruit and vegetable intake and frequent fast-food consumption were associated with lower odds of overweight/obesity.<br>d) Insufficient daily dairy intake was associated with lower odds of thinness.<br>e) The findings addressed the study objective of examining associations between dietary behaviours and nutritional status among Malaysian adolescents. |
| Limitations | 19 | Discuss limitations of the study, taking into account sources of potential bias or imprecision. Discuss both direction and magnitude of any potential bias | 15 | a) The cross-sectional study design limits causal inference between dietary behaviours and nutritional status outcomes and may introduce reverse causality.<br>b) Dietary behaviours were self-reported and may be subject to recall bias and reporting bias, particularly underreporting among overweight or obese adolescents.<br>c) Residual confounding from unmeasured factors, such as total energy intake and other lifestyle behaviours, may have influenced the observed associations.<br>d) Some unexpected inverse associations, such as inadequate fruit and vegetable intake and frequent fast-food consumption being associated with lower odds of |

| | | | | |
|---|---|---|---|---|
| | | | | overweight/obesity, may reflect reporting bias or unmeasured behavioural factors.<br>e) Although the proportion of missing data was low, exclusion of participants with missing data through complete-case analysis may have introduced minor selection bias. |
| Interpretation | 20 | Give a cautious overall interpretation of results considering objectives, limitations, multiplicity of analyses, results from similar studies, and other relevant evidence | 11-14 | a) The findings highlight the coexistence of undernutrition and overnutrition among Malaysian adolescents together with a high prevalence of unhealthy dietary behaviours.<br>b) Daily carbonated soft drink intake was associated with higher odds of overweight/obesity, supporting concerns regarding sugar-sweetened beverage consumption among adolescents.<br>c) Unexpected inverse associations observed for inadequate fruit and vegetable intake and frequent fast-food consumption should be interpreted cautiously due to possible reporting bias, reverse causality, and residual confounding.<br>d) Overall, the findings underscore the complexity of adolescent dietary behaviours and support the need for strengthened nutrition education and healthier food environments among Malaysian adolescents. |
| Generalisability | 21 | Discuss the generalisability (external validity) of the study results | 3-4 | a) The study utilised a nationwide sample of Malaysian school-based adolescents aged 13–18 years selected using a two-stage stratified cluster sampling design.<br>b) Therefore, the findings are likely generalisable to Malaysian school-based adolescents aged 13–18 years.<br>c) However, the findings may not be generalisable to adolescents who were not attending school. |
| **Other information** | | | | |
| Funding | 22 | Give the source of funding and the role of the funders for the present study and, if applicable, for the original study on which the present article is based | | Not applicable |

*Give information separately for cases and controls in case-control studies and, if applicable, for exposed and unexposed groups in cohort and cross-sectional studies.

**Note:** An Explanation and Elaboration article discusses each checklist item and gives methodological background and published examples of transparent reporting. The STROBE checklist is best used in conjunction with this article (freely available on the Web sites of PLoS Medicine at http://www.plosmedicine.org/, Annals of Internal Medicine at http://www.annals.org/, and Epidemiology at http://www.epidem.com/). Information on the STROBE Initiative is available at www.strobe-statement.org.
